# Autophagy Induction Is a Tor- and Tp53-Independent Cell Survival Response in a Zebrafish Model of Disrupted Ribosome Biogenesis

**DOI:** 10.1371/journal.pgen.1003279

**Published:** 2013-02-07

**Authors:** Yeliz Boglev, Andrew P. Badrock, Andrew J. Trotter, Qian Du, Elsbeth J. Richardson, Adam C. Parslow, Sebastian J. Markmiller, Nathan E. Hall, Tanya A. de Jong-Curtain, Annie Y. Ng, Heather Verkade, Elke A. Ober, Holly A. Field, Donghun Shin, Chong H. Shin, Katherine M. Hannan, Ross D. Hannan, Richard B. Pearson, Seok-Hyung Kim, Kevin C. Ess, Graham J. Lieschke, Didier Y. R. Stainier, Joan K. Heath

**Affiliations:** 1 Colon Molecular and Cellular Biology Laboratory, Ludwig Institute for Cancer Research, Melbourne-Parkville Branch, Melbourne, Victoria, Australia; 2 Department of Surgery, Faculty of Medicine, Dentistry and Health Sciences, University of Melbourne, Melbourne, Victoria, Australia; 3 Department of Biochemistry and Biophysics, University of California San Francisco, San Francisco, California, United States of America; 4 Peter MacCallum Cancer Centre, East Melbourne, Victoria, Australia; 5 Department of Neurology, Vanderbilt University Medical Centre, Nashville, Tennessee, United States of America; 6 Australian Regenerative Medicine Institute, Monash University, Clayton, Victoria, Australia; Stowers Institute for Medical Research, United States of America

## Abstract

Ribosome biogenesis underpins cell growth and division. Disruptions in ribosome biogenesis and translation initiation are deleterious to development and underlie a spectrum of diseases known collectively as ribosomopathies. Here, we describe a novel zebrafish mutant, *titania* (*tti^s450^*), which harbours a recessive lethal mutation in *pwp2h*, a gene encoding a protein component of the small subunit processome. The biochemical impacts of this lesion are decreased production of mature 18S rRNA molecules, activation of Tp53, and impaired ribosome biogenesis. In *tti^s450^*, the growth of the endodermal organs, eyes, brain, and craniofacial structures is severely arrested and autophagy is up-regulated, allowing intestinal epithelial cells to evade cell death. Inhibiting autophagy in *tti^s450^* larvae markedly reduces their lifespan. Somewhat surprisingly, autophagy induction in *tti^s450^* larvae is independent of the state of the Tor pathway and proceeds unabated in Tp53-mutant larvae. These data demonstrate that autophagy is a survival mechanism invoked in response to ribosomal stress. This response may be of relevance to therapeutic strategies aimed at killing cancer cells by targeting ribosome biogenesis. In certain contexts, these treatments may promote autophagy and contribute to cancer cells evading cell death.

## Author Summary

Autophagy is an act of self-preservation whereby a cell responds to stressful conditions such as nutrient depletion and intense muscular activity by digesting its own cytoplasmic organelles and proteins to fuel its longer-term survival. An understanding of the wide spectrum of physiological stimuli that can trigger this beneficial cellular mechanism is only just starting to emerge. However, this process also has a negative side, since autophagy is exploited in certain pathological conditions, including cancer, to extend the lifespan of cells that would otherwise die. Our analysis of a new zebrafish mutant, *titania* (*tti^s450^*), with defective digestive organs and abnormal craniofacial structure, sheds further light on the physiological and pathological ramifications of autophagy. In (*tti^s450^*), an inherited mutation in a gene required for ribosome production provides a powerful stimulus to autophagy in affected tissues, allowing them to evade cell death. The phenotypic consequences of impaired ribosome biogenesis in our zebrafish model are reminiscent of some of the clinical features associated with a group of human syndromes known as ribosomopathies.

## Introduction

The generation of new ribosomes is the most energy-consuming process in the cell [1]. It requires the coordinated transcription and maturation of 4 different ribosomal RNA (rRNA) molecules and 70 small nucleolar RNAs (snoRNAs) together with the synthesis of approximately 80 ribosomal proteins (RPs) and an additional 170 associated proteins [2]. The regulation of this complex, multi-step process is the major factor determining the potential of a cell to grow and divide [3]. In times of nutrient availability and/or hormonal and growth factor signalling, the onset of ribosome biogenesis is tightly coupled to the translational requirements of a rapidly proliferating cell. In contrast, ribosome biogenesis is down-regulated to conserve energy and restrict unwarranted cell growth and division when the cellular environment is nutrient poor or challenged by harmful stimuli such as hypoxia, reactive oxygen species or genotoxic stress. Inherited impairment mutations in genes that encode components of the ribosome biogenesis machinery or ribosome structure underlie a number of human syndromes, collectively known as ribosomopathies, with a broad range of clinical phenotypes [4]. There is a growing appreciation that sporadically acquired mutations in genes that contribute to ribosome function also increase susceptibility to human cancer, particularly leukemia and lymphoma, although the precise mechanisms involved are only just beginning to emerge [5].

The process of human ribosome biogenesis initiates in the nucleolus with the transcription by RNA polymerase (Pol) I of a 45S pre-rRNA precursor (35S in yeast), which contains the mature 28S, 18S and 5.8S rRNAs interspersed by spacer sequences. A series of processing and chemical modification events mediated by discrete multiprotein/RNA complexes known as the 90S, 66S and 43S pre-ribosomal particles generate the mature 18S, 28S and 5.8S species, respectively and assembles them into the 40S and 60S ribosomal subunits prior to their export from the nucleus to the cytoplasm where they associate to form the functional 80S ribosomes [6]. In yeast, the 90S particle, also known as the small-subunit processome, has been shown to be strictly required for the production of 40S ribosomal subunits containing 18S rRNA [7].

One of the mechanisms through which ribosome biogenesis is coupled to cell growth and proliferation is the Target of rapamycin (Tor) pathway, which is activated by cell surface growth factor and insulin receptors and other growth promoting sensors that detect when nutrients such as amino acids are plentiful. Activation of the Tor pathway stimulates the phosphorylation of S6 kinase (S6K) and 4E-Binding Protein 1 (4EBP1), which regulate ribosome biogenesis and mRNA translation [8], [9]. Activation of Tor also inhibits macroautophagy (hereafter referred to as autophagy), an evolutionarily conserved process that provides a survival mechanism during periods of cell starvation by promoting intracellular recycling of organelles, such as mitochondria and ribosomes [10], [11].

Autophagy describes a complex multi-step process whereby cells sequester a portion of their cytoplasm inside double-membrane vesicles called autophagosomes, which then fuse with lysosomes to form autolysosomes [12]. Inside these vesicles, the captured material, together with the inner membrane, is digested and the released nutrients are recycled. In metazoa, autophagy mediates the catabolic turnover of malfunctioning, damaged or superfluous proteins and organelles to maintain cellular homeostasis during development and in adult life [13]. It is activated in response to multiple forms of cellular stress, including nutrient deprivation, endoplasmic reticulum (ER) stress, accumulation of reactive oxygen species, DNA damage, invasion by intracellular pathogens and intense exercise [14], [15]. Some of these triggers induce autophagy through activation of Tumour protein 53 (Tp53), which increases the expression of the β1 and β2 subunits of AMP-activated protein kinase (AMPK), an evolutionarily conserved sensor of cellular energy levels [16]. AMPK responds to reductions in the ratio of ATP:AMP nucleotides by phosphorylating multiple targets with functions related to energy metabolism, including the Tuberous sclerosis complex (Tsc) protein, Tsc2 and Raptor. These phosphorylation events indirectly inhibit the Torc1 complex, which in its active state inhibits autophagy by negatively regulating the protein kinase, Ulk1 (mammalian orthologue of yeast Atg1). Ulk1, together with Atg13, Fip200 and Atg101, are the key components of a complex that initiates mammalian autophagosome formation [17], [18]. Recent work proposes that AMPK may also induce autophagy independently of Torc1 inhibition by directly phosphorylating Ulk1 [19]–[21]. However, a clear understanding of the AMPK-Ulk1-Torc1 network is yet to emerge [22].

In this study, we employed a zebrafish intestinal mutant, *titania^s450^ (tti^s450^)*, as an *in vivo* model to examine the connection between rRNA processing and autophagy. *tti^s450^* was identified on the basis of its hypoplastic intestinal morphology at 96 hours post-fertilization (hpf) in a focused ENU mutagenesis screen designed to identify mutants with defects in the size and morphology of the endoderm-derived organs [23]. Using positional cloning we identified *periodic tryptophan protein 2 homologue* (*pwp2h*) as the mutated gene in *tti^s450^*. In yeast, Pwp2 has been shown to be an essential scaffold component of the 90S pre-ribosomal particle, facilitating the binding of proteins such as the U3 snoRNP to the 5′ end of the 35S rRNA precursor [24]. Depletion of Pwp2 in yeast cells results in reduced production of mature 18S rRNA and 40S ribosomal subunits [24], [25]. In agreement with these results, we show that zebrafish Pwp2h plays a conserved role in rRNA processing and ribosome biogenesis. Moreover, we use this *in vivo* model system to demonstrate a connection between rRNA processing and autophagy which has, to our knowledge, been hitherto unappreciated.

## Results

### *tti^s450^* larvae exhibit defects in intestinal, liver, pancreas, and craniofacial development

**Figure 1:**
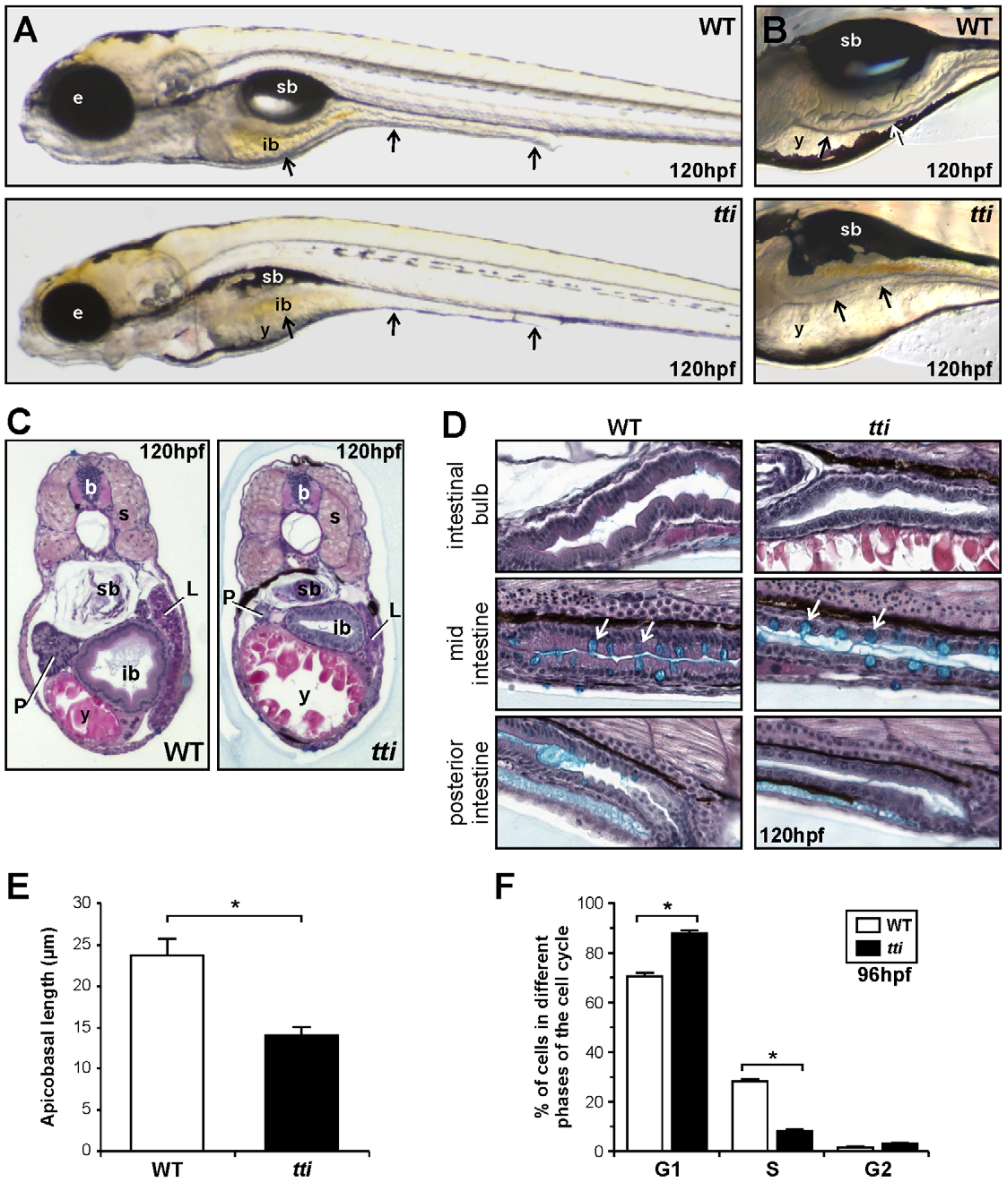
The *tti^s450^* phenotype encompasses craniofacial defects, smaller endodermal organs, and microphthalmia. (A, B) Differential interference contrast (DIC) images of WT and *tti^s450^* larvae at 120 hpf. (A) The black arrows indicate, from left to right, the 3 regions of the intestine: the intestinal bulb, mid-intestine and posterior intestine. (B) The intestinal epithelium in WT larvae is extensively folded *(upper panel)* and is thinner and unfolded in *tti^s450^* larvae *(bottom panel)*. In *tti^s450^*, yolk resorption is incomplete and the swim bladder does not inflate. Microphthalmia is evident and the head is slightly smaller and misshapen. (C, D) Transverse (C) and sagittal (D) histological sections of WT and *tti^s450^* larvae at 120 hpf stained with alcian blue periodic acid-Schiff reagent. The anterior part of the intestine (intestinal bulb) is expanded and the epithelium is elaborated into folds in WT larvae *(C, left panel)*. In *tti^s450^* the intestinal bulb, liver and pancreas are smaller than in WT and the epithelium is relatively thin and flat (C, *right panel*). (D) The intestinal epithelial cells of the entire intestinal tract are columnar in shape in WT larvae (*left panels*) and are cuboidal in *tti^s450^ (right panels)*. Goblet cells containing acidic mucins (turquoise staining) are present in approximately equal numbers (white arrows) in the WT and *tti^s450^* mid-intestine. sb, swim bladder; b, brain; ib, intestinal bulb; y, yolk; e, eye; s, somite; P, pancreas; L, liver; (E) The average apicobasal length of the IECs in the intestinal bulb region of *tti^s450^* larvae at 120 hpf is approximately half that of WT IECs. Measurements were performed on 10 cells in 3 independent sections. (F) Fluorescent activated cell sorting analysis of the cell cycle in cells derived from the GFP-positive, endoderm derived organs (liver, pancreas, intestine) of *tti* ^s450^ and WT larvae on the gutGFP background at 96 hpf. Data are represented as the mean +/− SD (n = 3), *p<0.05.

*tti^s450^* is one of several intestinal mutants identified in an ENU mutagenesis screen (the Liver^plus^ screen) conducted on a transgenic line of zebrafish (*Tg(XlEef1a1:GFP)^s854^*) harbouring a GFP transgene (“gutGFP”) expressed specifically in the digestive organs [23], [26], [27]. Abnormalities in the gross morphology of *tti^s450^* larvae are first detectable at 72 hpf and became more severe with time. At 120 hpf, the wildtype (WT) intestinal epithelium exhibits a columnar morphology and starts to elaborate folds; in contrast, the intestinal epithelium in *tti^s450^* remains thin and unfolded (Figure 1A and 1B). *tti^s450^* larvae also exhibit smaller eyes (microphthalmia), a smaller, misshapen head, an uninflated swim bladder and impaired yolk absorption (Figure 1A). At 120 hpf, the *tti^s450^* pancreas and liver are both substantially smaller than in WT (Figure 1C).

By 120 hpf, the rostral intestine (intestinal bulb region) in *tti^s450^* larvae is markedly smaller than in WT and the intestinal epithelial cells (IECs) are cuboidal rather than columnar in shape (Figure 1C, 1D). The intestinal lumen appears clear of cellular debris. Cells in the mid and posterior intestine are also smaller and less polarized than in WT (Figure 1D). The mean apicobasal height of the cells in the intestinal bulb region of *tti^s450^* larvae is approximately 40% less than that in WT (Figure 1E). However, cellular differentiation is not inhibited as similar numbers of mucin-producing goblet cells are found in the mid-intestinal region of *tti^s450^* larvae as in WT (Figure 1D).

The reduction in cell size is accompanied by changes in the proportion of cells in different phases of the cell cycle. At 72 hpf, the intestinal epithelium is the most rapidly proliferating tissue in the zebrafish embryo [28], [29]. Using BrdU incorporation analysis, we detected fewer *tti^s450^* IECs in *S* phase than WT IECs (Figure S1A, S1B). Fluorescent activated cell sorting (FACS) of cells disaggregated from WT and *tti^s450^* larvae carrying the gutGFP transgene allowed us to analyze the proliferation of cells derived specifically from the liver, pancreas and intestine. We observed a significant accumulation of *tti^s450^* cells in the *G1* phase of the cell cycle at 96 hpf (88% in *tti^s450^* compared to 70% in WT) and a corresponding reduction of *tti^s450^* cells in *S* phase (8% in *tti^s450^* compared to 28% in WT). No significant difference in the number of cells in *G2* was observed (Figure 1F).

The *tti^s450^* phenotype is completely penetrant, and the animals die at 8–9 days post-fertilization (dpf). Heterozygous *tti^s450^* carriers are phenotypically indistinguishable from WT siblings.

### *tti^s450^* harbours a mutation in *pwp2h*

**Figure 2:**
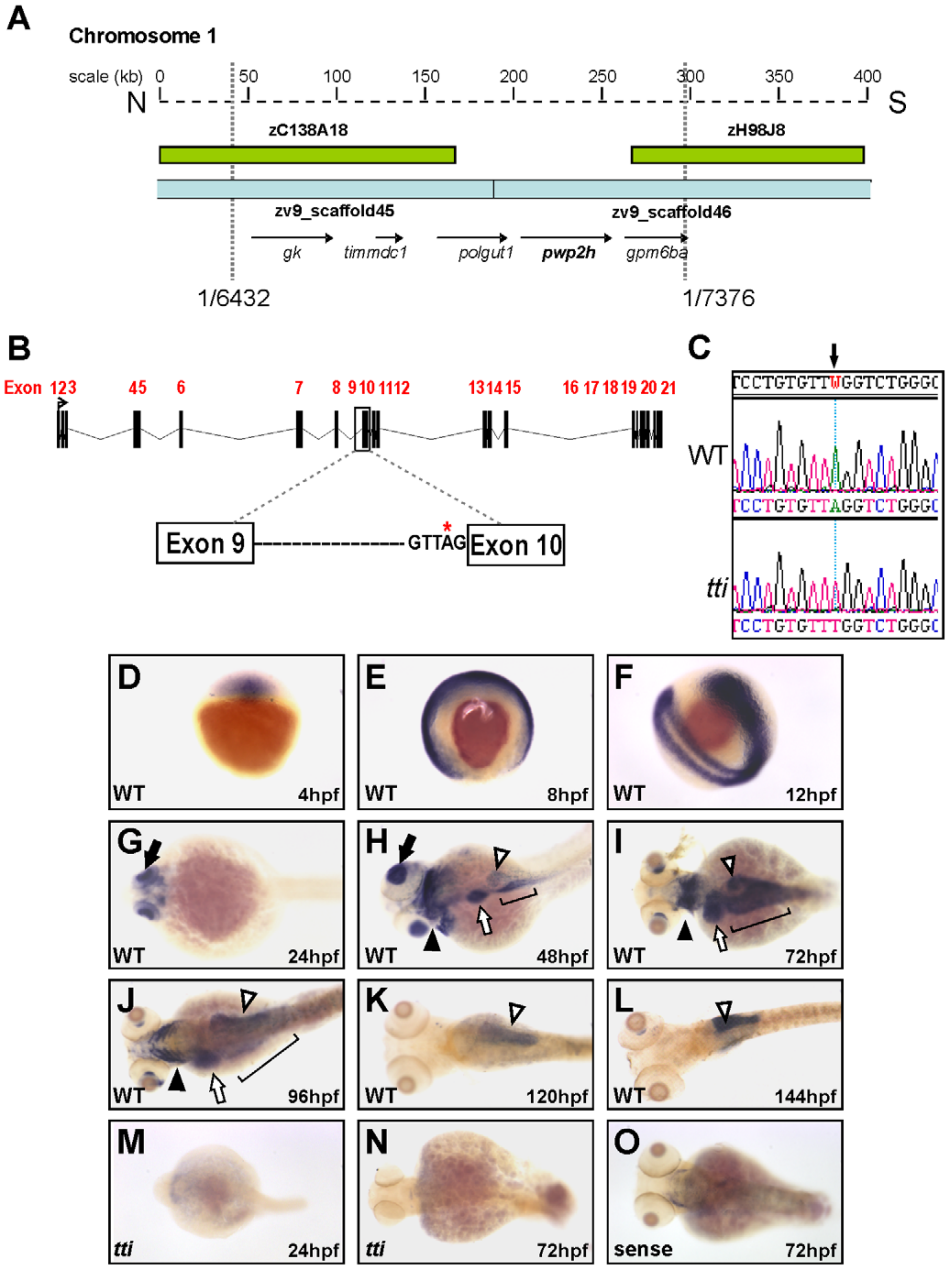
Positional cloning reveals that *pwp2h* is the mutated gene in *tti^s450^*. (A) Physical map of chromosome 1 in the region encompassing the *tti^s450^* locus. Analysis of recombinants from 7376 meioses narrowed the genetic interval containing the mutation to a region flanked by 2 BACs (green boxes) and encompassed by 2 scaffolds zv945445 and zv945446 (blue bars) containing 5 genes (arrows). (B) Schematic representation of the *pwp2h* gene and the location of the sequence variation in intron 9. (C) The nucleotide sequence of *pwp2h* cDNA from *tti^s450^* larvae contains an A→T transversion. Wholemount *in situ* hybridization (WISH) reveals the *pwp2h* mRNA expression pattern from 4–144 hpf in WT larvae (D–L). *pwp2h* expression is ubiquitous from 4–12 hpf (D–F), restricted to the retina at 24 hpf (G; *black arrow*) and encompasses the pharyngeal cartilages *(black arrowhead)*, liver *(white arrow)*, intestine *(bracket)* and pancreas *(white arrowhead)* at 48 hpf (H), 72 hpf (I) and 96 hpf (J). From 120–144 hpf *pwp2h* expression is restricted to the pancreas (K–L; *white arrowhead*). *pwp2h* expression is barely detectable at 24 hpf (M) and 72 hpf (N) in *tti^s450^* larvae. Staining is absent in the sense control at 72 hpf (O) and at all other time points (data not shown).

We identified the mutated gene responsible for the abnormal digestive organ development in *tti^s450^* by mapping the *tti^s450^* locus to a 260-kilobase interval on chromosome 1 encompassing 5 genes (Figure 2A). One of these genes, *pwp2h*, comprises 21 exons spanning 2928 base pairs (Figure 2B) and encodes a protein of 937 amino acids containing 13 WD-40 repeat domains. WD-40 repeats generally serve as platforms for the assembly of proteins in multi-protein complexes and are conserved from yeast to mammals. We identified an A to T base change in the conserved splice acceptor site in intron 9 of *pwp2h* in *tti^s450^* mutants (Figure 2C) resulting in utilization of a cryptic splice site 11 bp upstream of exon 10, thereby generating a frame-shift and nonsense mutation in codon 421 (Figure S2A) and truncating the Pwp2h protein in the seventh WD domain (Figure S3).

The *tti* phenotype is recapitulated by microinjection of 1–4 cell zebrafish embryos with an antisense morpholino oligonucleotide targeted to *pwp2h* mRNA (Figure S2B, S2C). That mutant *pwp2h* is responsible for the *tti^s450^* phenotype was confirmed by non-complementation with an independent allele of *pwp2h*, *tti^s927^* (Figure S2D–S2G). *tti^s927^* was identified in an ENU mutagenesis screen (the 2-CLIP screen) [30] conducted on the (*ins*:*dsRed*)*^m1081^*;*Tg*(*fabp10*:*dsRed*;*ela3l*:*GFP*)*^gz12^* transgenic background [31] to facilitate assessment of pancreas and liver development. *tti* ^s927^ harbours a missense mutation in *pwp2h*: a T to A transversion in exon 5 (Figure S2H) resulting in the replacement of a valine with glutamic acid (Figure S2I) in the second WD-40 domain (Figure S3). The phenotypes of *tti^s450^* and *tti* ^s927^ larvae are essentially indistinguishable.

### The *pwp2h* mRNA expression pattern delineates the tissues that are abnormal in *tti^s450^*

In order to assess the expression pattern of *pwp2h* during zebrafish embryogenesis, we performed wholemount *in situ* hybridization (WISH). In WT embryos *pwp2h* mRNA is ubiquitously expressed between 4–12 hpf and then becomes restricted to the brain and eyes at 24 hpf (Figure 2D–2G). By 48 hpf *pwp2h* mRNA is expressed in the pharyngeal cartilages and primitive gut, including the liver and pancreas anlagen (Figure 2H). By 72 hpf expression in the eye is largely extinguished and restricted to the pharyngeal cartilages, liver, intestine and pancreas (Figure 2I). By 96 hpf, *pwp2h* expression in the intestine is diminishing but is sustained in the pharyngeal cartilages, liver and pancreas (Figure 2J). By 120–144 hpf, the pancreas is the only tissue in which *pwp2h* mRNA is detected (Figure 2K, 2L). Expression of *pwp2h* is absent in *tti^s450^* embryos from 24 hpf onwards (Figure 2M, 2N) indicating that upon exhaustion of maternally deposited supplies of WT *pwp2h* mRNA, the zygotically expressed mutant mRNA probably undergoes nonsense-mediated decay (NMD). These expression data are consistent with the eye, brain, pharyngeal cartilages and digestive organs being the most severely affected organs in *tti^s450^* larvae.

### *pwp2h* deficiency leads to impaired ribosome biogenesis in *tti^s450^* larvae

**Figure 3:**
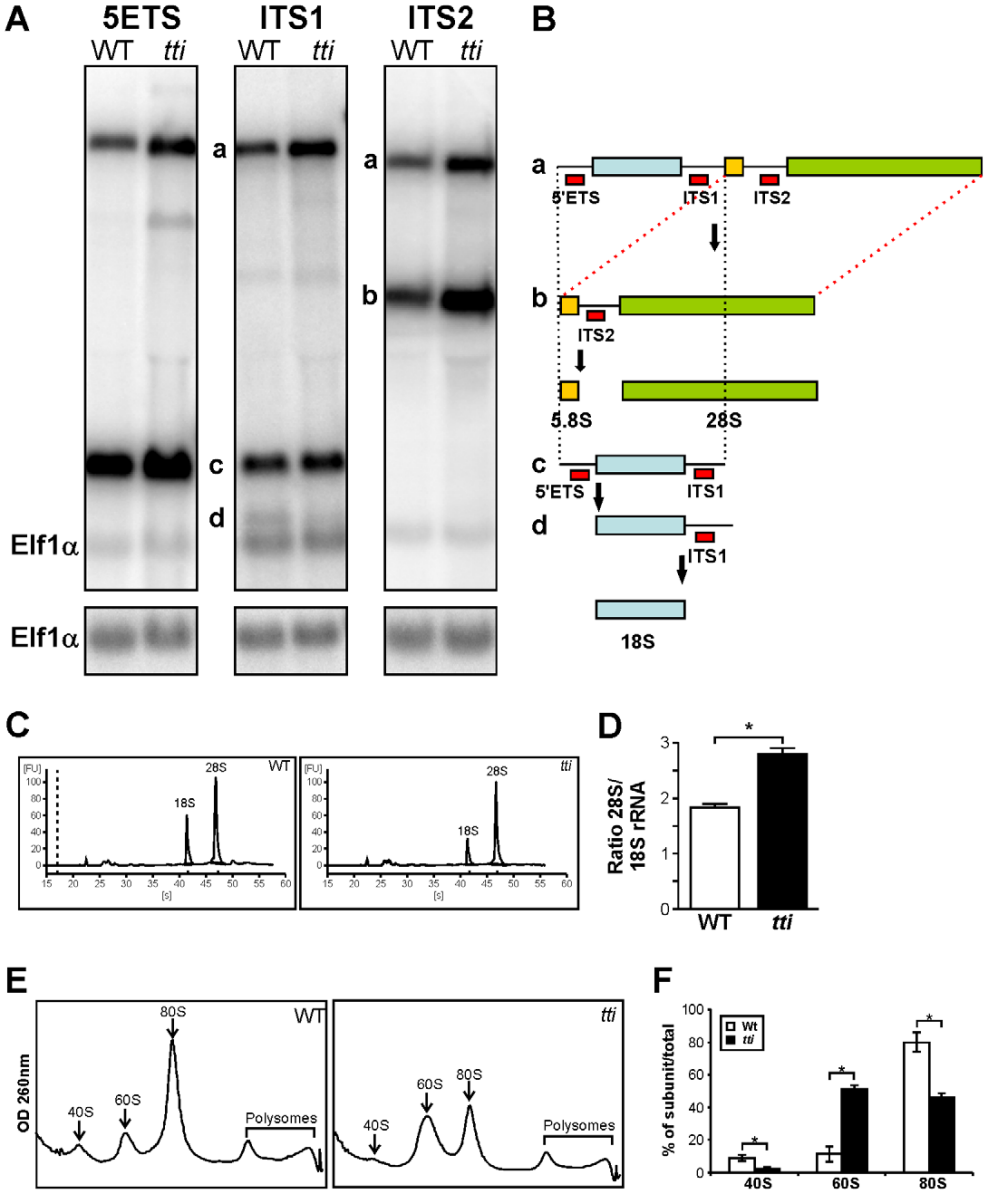
*tti^s450^* larvae display defects in ribosome biogenesis. (A) Northern analysis of RNA isolated from WT and *tti^s450^* larvae at 120 hpf using 5′ETS, ITS1, and ITS2 probes to detect precursor forms of rRNA. Elf1α is a loading control. a–d correspond to the rRNA intermediates depicted in Figure 3B. (B) Schematic diagram showing the rRNA processing pathway in zebrafish [60]. The sites of hybridization of the 5′ETS, ITS1 and ITS2 probes are indicated. (C) Representative E-Bioanalyser analysis of total RNA isolated from WT and *tti^s450^* larvae at 120 hpf demonstrates a reduction in the 18S peak in *tti^s450^* larvae resulting in an elevated 28S/18S rRNA ratio in *tti^s450^* (D). Graphical representation of the experiment shown in C. Data are represented as mean +/− SD (n = 5). (E) Representative polysome fractionation analysis performed on WT and *tti^s450^* larvae at 96 hpf demonstrates reduced levels of 40S ribosomal subunits and 80S monosomes and an increase in free 60S subunits in *tti^s450^* larvae compared to WT. (F) Graphical representation of the experiment shown in E. Data are represented as mean +/− SD (n = 5) *p<0.05.

In all species, rRNA is transcribed as a large pre-rRNA transcript which undergoes a series of enzymatic cleavage steps within the nucleolus by large ribonucleoprotein complexes to produce mature 18S, 28S and 5.8S rRNAs (Figure 3B). To investigate rRNA processing in *tti^s450^* larvae, we conducted Northern blot analysis (Figure 3A) using probes designed to hybridize to the external (5′ETS) and internal-transcribed (ITS1 and ITS2) spacer regions of zebrafish 45S pre-rRNA (Figure 3B). These probes detect the full-length rRNA precursor and all intermediate species but not the fully mature forms of rRNA. This analysis revealed a 2.5 fold accumulation of the full-length precursor ‘a’ in *tti^s450^* and an accumulation of the intermediates ‘b’ and ‘c’ (4.6 fold and 1.3 fold, respectively). These observations are consistent with a block in the processing of the full-length rRNA precursor. We also noted a 2.6 fold decrease in *tti^s450^* larvae in the level of ‘d’, the immediate precursor of 18S rRNA (Figure 3A). Furthermore, E-bioanalyser analysis revealed a marked reduction in the production of mature 18S rRNA in *tti^s450^* larvae (Figure 3C); however, the production of mature 28S rRNA was unaffected (Figure 3C). These changes altered the ratio of 28S/18S rRNA in *tti^s450^* larvae, which is 2.8 at 120 hpf, compared to 1.8 in WT (Figure 3D).

To investigate the impact of Pwp2h deficiency on ribosome formation, we prepared extracts of WT and *tti* zebrafish larvae at 96 hpf and fractionated the ribosomal subunits on sucrose density gradients (Figure 3E). The areas under the peaks corresponding to the 40S subunits and 80S monosomes in *tti^s450^* lysates are markedly smaller compared to those in WT (reduced approximately 4 fold and 2-fold, respectively). Meanwhile, the area under the peak corresponding to the 60S subunits is increased by approximately 4.5 fold (Figure 3F). Collectively, these data are consistent with Pwp2h deficiency primarily impacting on 40S subunit formation.

### Intestinal epithelial cells in *tti^s450^* larvae undergo autophagy

**Figure 4:**
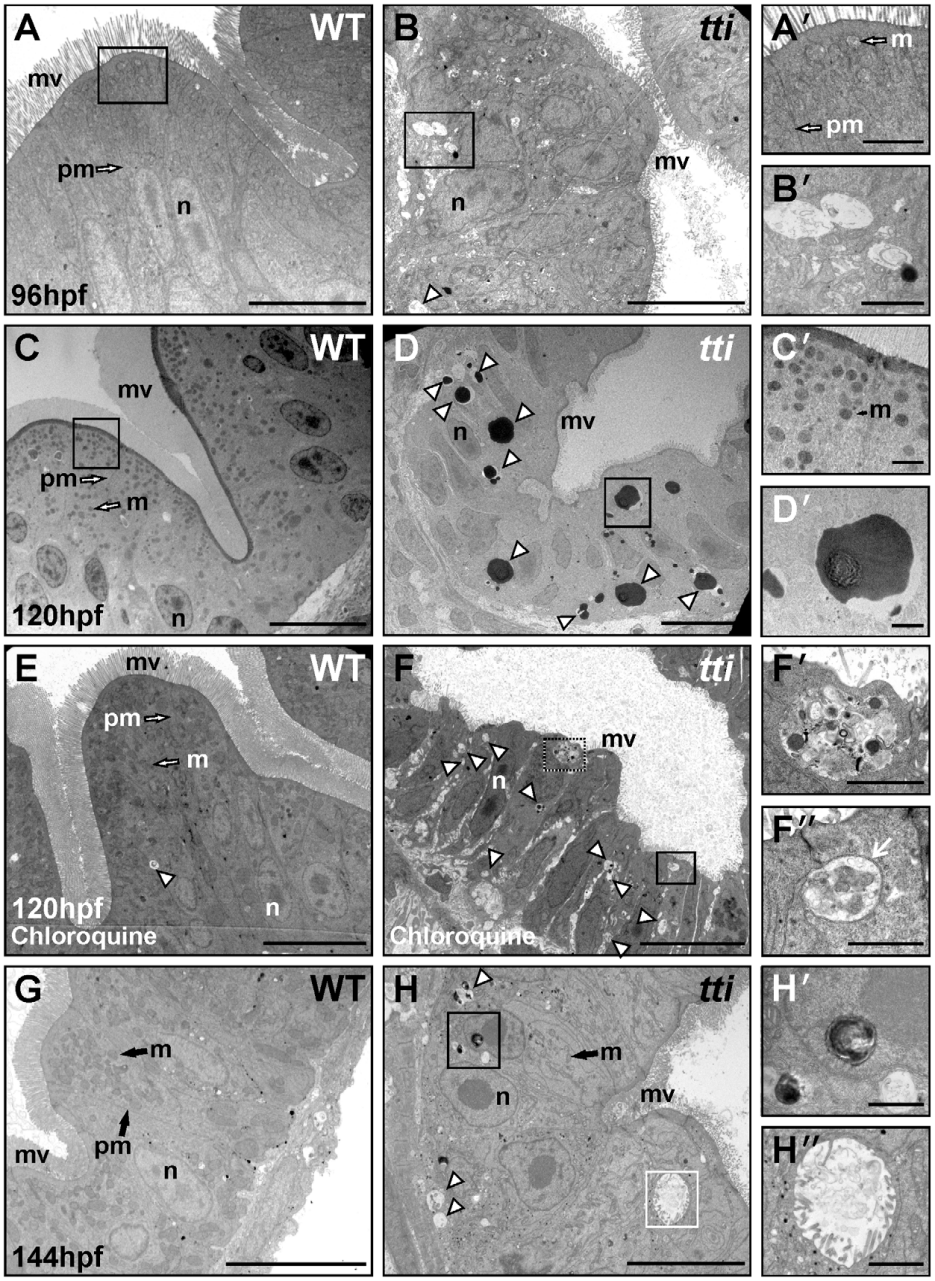
The intestinal epithelial cells (IECS) in *tti^s450^* larvae contain autophagosome- and autolysome-like structures. (A–H) Transmission electron micrographs of WT and *tti^s450^* larvae at 96 hpf (A, B), 120 hpf (C–F) and 144 hpf (G, H). Sections are transverse through the yolk in the region of the intestinal bulb. WT IECs demonstrate well-developed apicobasal polarity as evidenced by basally positioned nuclei (n) and the elaboration of microvilli (mv) projecting from the apical surface into the intestinal lumen. Mitochondria (m) are abundant and plasma membranes (pm) are well defined. The intestinal epithelium in *tti^s450^* is highly disorganized, with shorter and relatively sparse apical microvilli compared to WT. Vesicles resembling autophagosomes (*white arrowhead* in B) are present in the intestinal epithelial cells of *tti^s450^* larvae (B′ [boxed area in B], H″ [boxed area in H]) but not in WT (A, A′ [boxed area in A] and G). At 120 hpf, electron-dense structures, likely to correspond to autolysosomes, are present in *tti^s450^* larvae (*white arrowheads* in D, D′ [boxed area in D]), but not WT (C, C′ [boxed area in C]). When *tti^s450^* larvae are treated with chloroquine to block the fusion of autophagosomes with lysosomes, the electron-dense structures are no longer apparent at 120 hpf; instead vesicles more typical of autophagosomes are found (*white arrowheads* in F). The boxed areas in F (F′ and F″) show vesicles containing debris, including one (white arrow in F″), with a clear double membrane. Scale bars = 10 µm (A–H) and 1 µm (all insets).

To determine the impact of impaired ribosome biogenesis at the ultrastructural level, we used transmission electron microscopy (TEM) (Figure 4A–4H). While WT intestinal epithelium is folded and the cells exhibit apicobasal polarity and a highly elaborated apical brush border (Figure 4A, 4C, 4E, 4G), IECs in *tti^s450^* are smaller and the microvilli are shorter and relatively sparse (Figure 4B, 4D, 4F, 4H). The *tti^s450^* nuclei contain prominent condensed nucleoli, suggesting ribosomal stress [32]. Also conspicuous at 96 hpf in the IECs of *tti^s450^* larvae, but essentially absent in WT, are cytoplasmic vesicles containing debris (Figure 4B, 4B′). At 120 hpf, these structures are bigger in size and electron dense (Figure 4D, 4D′). At 144 hpf, vesicles more akin to those observed at 96 hpf are present (Figure 4H, 4H′, 4H″). Similar transient structures have been previously identified in cells undergoing autophagy. We therefore pursued the hypothesis that the cytoplasmic vesicles in *tti^s450^* larvae correspond to autophagosomes and autolysosomes: vesicles that sequester and digest organelles.

Autophagy is a dynamic process comprising autophagosome synthesis, delivery of autophagic substrates to lysosomes and substrate degradation in autolysosomes [10], [12]. In order to investigate whether the electron dense vesicles observed at 120 hpf (Figure 4D) correspond to autolysosomes, we exposed WT and *tti^s450^* larvae at 106 hpf for 14 h to chloroquine, an autophagy inhibitor that blocks the fusion of autophagosomes with lysosomes and thereby prevents digestion of the vesicle contents [33]. After chloroquine treatment few, if any, electron dense cytoplasmic vesicles (autolysosomes) are found in the intestinal epithelium of *tti^s450^* larvae (Figure 4F). Instead, the IECs in *tti^s450^* larvae contain vesicles more reminiscent of autophagosomes (Figure 4F, 4F′, 4F″). We counted >3 autophagosomes/cell (3.25±0.144, n = 60) in the IECs of *tti^s450^* larvae, compared to <1 (0.6±0.058, n = 60) in WT IECs. Thus chloroquine inhibition of autophagic flux results in a significantly higher number of autophagosome-like structures in *tti^s450^* larvae compared to WT.

**Figure 5:**
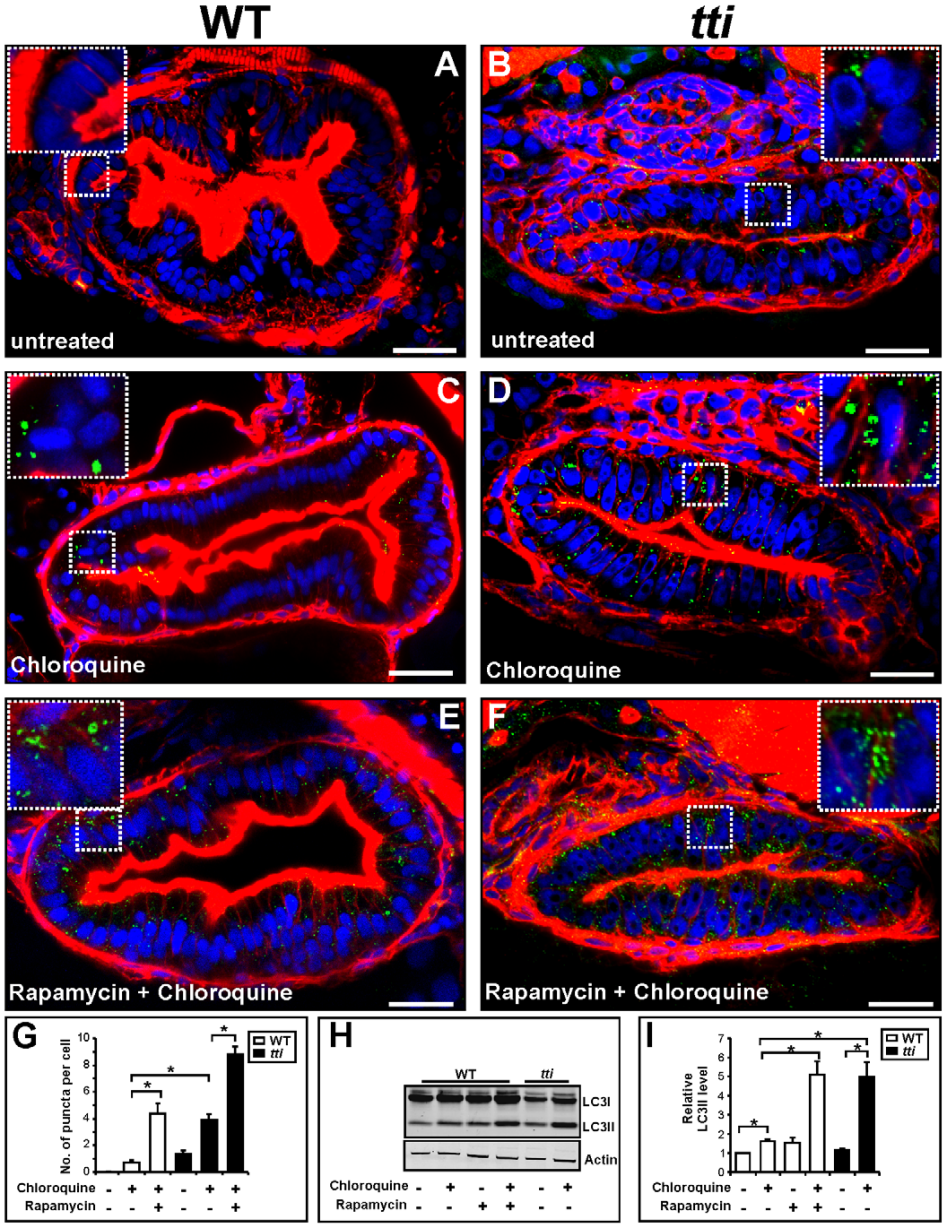
Comparable autophagic flux in the IECs of *tti^s450^* larvae and WT larvae treated with rapamycin. (A–F) Transverse sections (200 µm) through the intestinal bulb region of untreated WT (A) and *tti^s450^* (B) larvae at 120 hpf or larvae previously treated for 14 h with rapamycin and/or chloroquine (C–F) stained with rhodamine phalloidin to detect F-actin (*red*), Hoechst 33342 to detect DNA (*blue*) and the LC3B antibody to detect LC3II–containing autophagosomes (*green puncta*). (G) The numbers of autophagosomes are increased in chloroquine-treated WT and *tti^s450^* larvae compared to the corresponding untreated larvae. Chloroquine-treated *tti^s450^* larvae contain significantly more puncta than chloroquine-treated WT larvae and similar numbers to WT larvae treated with rapamycin and chloroquine. Rapamycin and chloroquine-treated *tti^s450^* larvae contain significantly more puncta per IEC than the IECs in chloroquine-treated *tti^s450^* larvae and chloroquine and rapamycin-treated WT larvae. Puncta were counted in 20 cells from 3 independent sections using Metamorph. (H) Representative Western blot analysis of whole cell lysates of WT and *tti^s450^* larvae (96 hpf) previously treated for 14 h with rapamycin (10 µM) and/or chloroquine (2.5 µM) using antibodies to LC3B and Actin (loading control). (I) Graphical representation of the data shown in H and two independent analyses. The LC3II signals were quantitated by densitometry. *tti^s450^* larvae treated with chloroquine contain more LC3II than their chloroquine-treated WT siblings and comparable levels to WT larvae treated with rapamycin and chloroquine. Data are represented as mean +/− SD, *p<0.05.

To investigate this further, we examined LC3 localisation in WT and *tti^s450^* larvae using wholemount immunocytochemistry (Figure 5A–5G). LC3, the mammalian orthologue of yeast Atg8, is a robust marker of autophagosomes. Upon induction of autophagy, the cytoplasmic form of LC3 (LC3I) is converted by cleavage and lipidation to a transient, autophagosomal membrane-bound form of LC3 (LC3II). Disrupting the fusion of autophagosomes with lysosomes with chloroquine prolongs the half-life of LC3II and facilitates the accumulation of LC3II-containing autophagosomes, which appear as punctate structures using LC3 immunocytochemistry. We observed more puncta in the IECs of chloroquine-treated WT larvae (Figure 5C) compared to untreated WT larvae (Figure 5A). Consistent with impaired ribosome biogenesis stimulating autophagy, we counted approximately 5 times more puncta in the IECs of chloroquine-treated *tti^s450^* larvae (Figure 5D) compared to the IECs of chloroquine-treated WT siblings (Figure 5C; compare 2^nd^ and 4^th^ bars in Figure 5G). We next exposed WT and *tti^s450^* larvae to rapamycin, which through its specific inhibition of Torc1 [34], [35] provides a powerful stimulus to autophagy in yeast, zebrafish and mice. We found that the number of puncta in WT larvae treated with rapamycin and chloroquine together (Figure 5E, 5G) was similar to the number of puncta in *tti^s450^* larvae treated with chloroquine alone (Figure 5D, 5G). Finally, treating *tti^s450^* larvae with rapamycin and chloroquine together (Figure 5F) resulted in more abundant puncta than in both chloroquine-treated *tti^s450^* larvae and rapamycin and chloroquine-treated WT larvae (Figure 5G). Upon Western blot analysis of whole larval lysates (Figure 5H, 5I), we found that LC3II levels in chloroquine-treated *tti^s450^* larvae were significantly higher than in chloroquine-treated WT larvae but not significantly different from those in WT larvae treated with rapamycin and chloroquine together (Figure 5I). Together these experiments demonstrate that the vesicles identified in the IECs of *tti^s450^* larvae are autophagosomes, and, to the best of our knowledge, provide the first evidence for a link between impaired ribosome biogenesis and autophagy.

To determine the extent of autophagy in *tti^s450^* larvae, we injected RNA encoding a mCherry-LC3 fusion protein into the yolk of 1–4 cell stage zebrafish embryos and evaluated the formation of puncta after prior treatment with chloroquine for 14 h at three time-points (Figure S4). At 72 hpf, abundant puncta are present in the eye (Figure S4B) and brain (Figure S4B′) of *tti^s450^* larvae compared to WT larvae (Figure S4A, S4A′). At this time-point, there are very few puncta in the digestive organs (Figure S4C, S4D). A similar picture was observed at 96 hpf (data not shown). At 120 hpf, the number of puncta in the brain (Figure S4F′) in *tti^s450^* larvae is now comparable to that observed in WT (Figure S4E′), while higher numbers of puncta are still found in the eye (Figure S4F). At 120 hpf there are more abundant puncta in the intestine and pancreas of *tti^s450^* larvae (Figure S4H) compared to these organs in WT (Figure S4E and S4G, respectively). This pattern of autophagy induction mirrors the tempero-spatial expression of *pwp2h* during zebrafish development, and is consistent with these tissues being the most affected by impaired ribosome biogenesis in *tti^s450^* larvae.

To determine whether autophagy is a specific response to impaired ribosome biogenesis, we conducted LC3 analysis of two additional zebrafish intestinal mutants, *setebos (set^s453^)* and *caliban (clbn^s846^)*, which exhibit phenotypes that are essentially indistinguishable from that of *tti^s450^* when viewed under the light microscope or upon histological analysis. Whereas *set^s453^* harbours a mutation in a gene which impairs 28S rRNA production and ribosome biogenesis (APB *et al.*, in preparation), the mutation in *clbn^s846^* lies in a gene encoding an essential mRNA splicing factor (SJM *et al.*, in preparation). We observed that *set^s453^* larvae, like *tti^s450^* larvae, contain higher LC3II levels compared to WT siblings in the presence of chloroquine (Figure S5A, S5B) and their IECs contain abundant autophagosome-like structures when analysed by TEM (data not shown). In contrast, the LC3II levels in *clbn^s846^* larvae are indistinguishable from those in WT siblings (Figure S5A, S5B) and the intestinal epithelium of *clbn^s846^* mutants do not contain autophagosomes or autolysosomes when inspected at the ultrastructural level (Figure S5C–S5H). These data suggest that the induction of autophagy in IECs is a specific response to impaired ribosome biogenesis, rather than a non-specific response to impaired cell growth.

### Autophagy induction in *tti^s450^* larvae prolongs their survival

**Figure 6:**
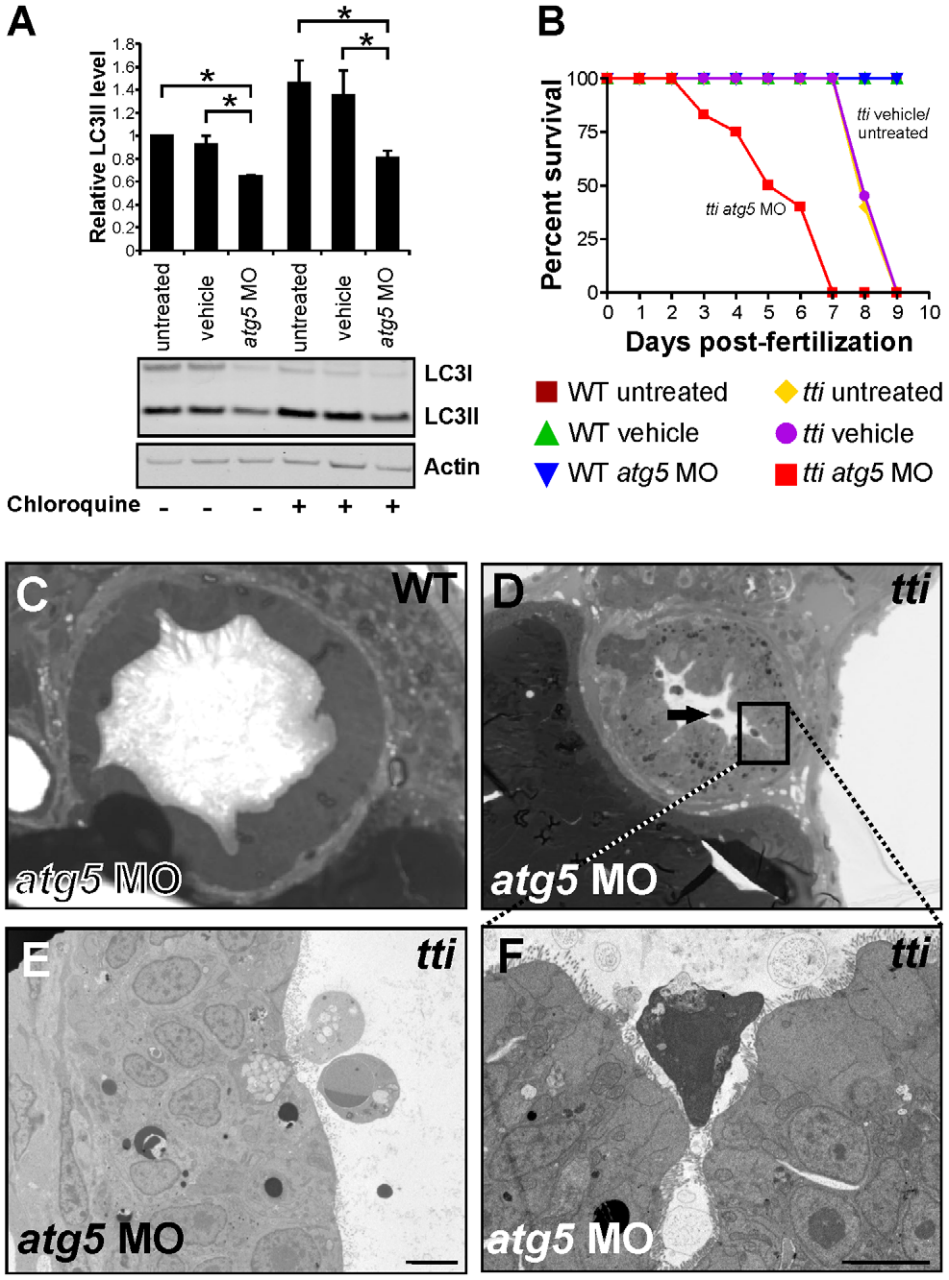
Disrupting autophagy in *tti^s450^* larvae results in the death of IECs and a reduced lifespan. (A) Western blot analysis of lysates of *tti^s450^* larvae (72 hpf) that had been injected at the 1–4 cell stage with an antisense morpholino oligonucleotide (MO) targeted to the start codon of *atg5* mRNA reveals decreased levels of LC3II compared to untreated and vehicle controls, both in the presence and absence of chloroquine. Data are represented as mean +/− SD, *p<0.05. (B) Survival curve of untreated WT and *tti^s450^* larvae compared to WT and *tti^s450^* larvae that had been injected at the 1–4 cell stage with vehicle or *atg5* MO (n>85 larvae per group). The lifespan of WT embryos/larvae is completely unaffected by injection with the *atg5* MO since all three groups of WT larvae (untreated, vehicle-treated and *atg5* MO-treated) progress normally through the first 10 days of development, when the experiment was terminated. The horizontal line represents untreated WT embryos (maroon squares), vehicle-injected WT embryos (green triangles) and *atg5* MO-injected WT embryos (blue triangles). In contrast, *tti^s450^* embryos respond to microinjection of the *atg5* MO by impaired survival. Whereas all untreated (yellow diamonds) or vehicle-injected (purple circles) *tti^s450^* larvae are still alive at 7 dpf, all the *atg5* MO-injected *tti^s450^* larvae are dead at this time-point (red squares). Indeed, 20% of the *atg5* MO-injected *tti^s450^* larvae have already succumbed by 3 dpf. (C–F) TEMs of WT (C) and *tti^s450^* larvae at 120 hpf (D–F), injected at the 1–4 cell stage with the *atg5*-targeted MO. Inhibiting autophagy in *tti^s450^* larvae results in the appearance of detached and shrunken IECs in the intestinal lumen (black arrow in D, E and F [boxed area in D]) but has no impact on WT IECs (C). Scale bars = 10 µm.

We followed the morphological changes in the intestinal epithelium and liver of *tti^s450^* larvae until 7 dpf, just before the larvae die at 8–9 dpf. At 7 dpf, the IECs are substantially smaller in *tti^s450^* larvae than in their WT counterparts and neither *tti^s450^* nor WT larvae contain detached cells in the intestinal lumen (Figure S6A–S6D). The *tti^s450^* IECs no longer contain conspicuous autophagosomes, though electron dense vesicles are present in abundance in adjacent liver cells (Figure S6E–S6F). To investigate the impact of inhibiting autophagy in *tti^s450^* larvae, we blocked autophagosome formation by injecting 1 ng of an antisense morpholino oligonucleotide (MO), which targets the translation start-site of *atg5* mRNA [36], into 1–4 cell stage embryos derived from pair-wise matings of heterozygous *tti^s450^* adults. At 72 hpf, uninjected, vehicle-injected and *atg5* MO-injected *tti^s450^* larvae were identified and subjected to LC3 analysis. We found significantly lower LC3II levels in the *atg5* MO-injected *tti^s450^* larvae compared to uninjected and vehicle-injected controls (Figure 6A). Moreover, from 72–120 hpf, we noticed that *atg5* MO-injected *tti^s450^* larvae start to develop oedema around the head, eye, heart and intestine (Figure S7D). As a consequence, 50% of *atg5* MO-injected *tti^s450^* larvae die by 5 dpf and all *atg5* MO-injected *tti^s450^* larvae are dead by 7 dpf (Figure 6B). This contrasts markedly with untreated or vehicle-injected *tti^s450^* larvae, which survive until 8–9 dpf (Figure 6B). The longevity of WT larvae injected with the *atg5* MO is not affected. Ultrastructural analysis at 120 hpf revealed detached, shrunken cells in the intestinal lumen of *atg5* MO-treated *ti^s450^* larvae (Figure 6D–6F) that were never seen in the intestinal lumen of *tti^s450^* larvae injected with vehicle or WT siblings injected with *atg5* MO (Figure 6C). Together these data demonstrate that autophagy extends the lifespan of *tti^s450^* larvae and prolongs the survival of IECs.

### Autophagy induction in *tti^s450^* larvae is independent of Tor pathway activity and p-RPS6

**Figure 7:**
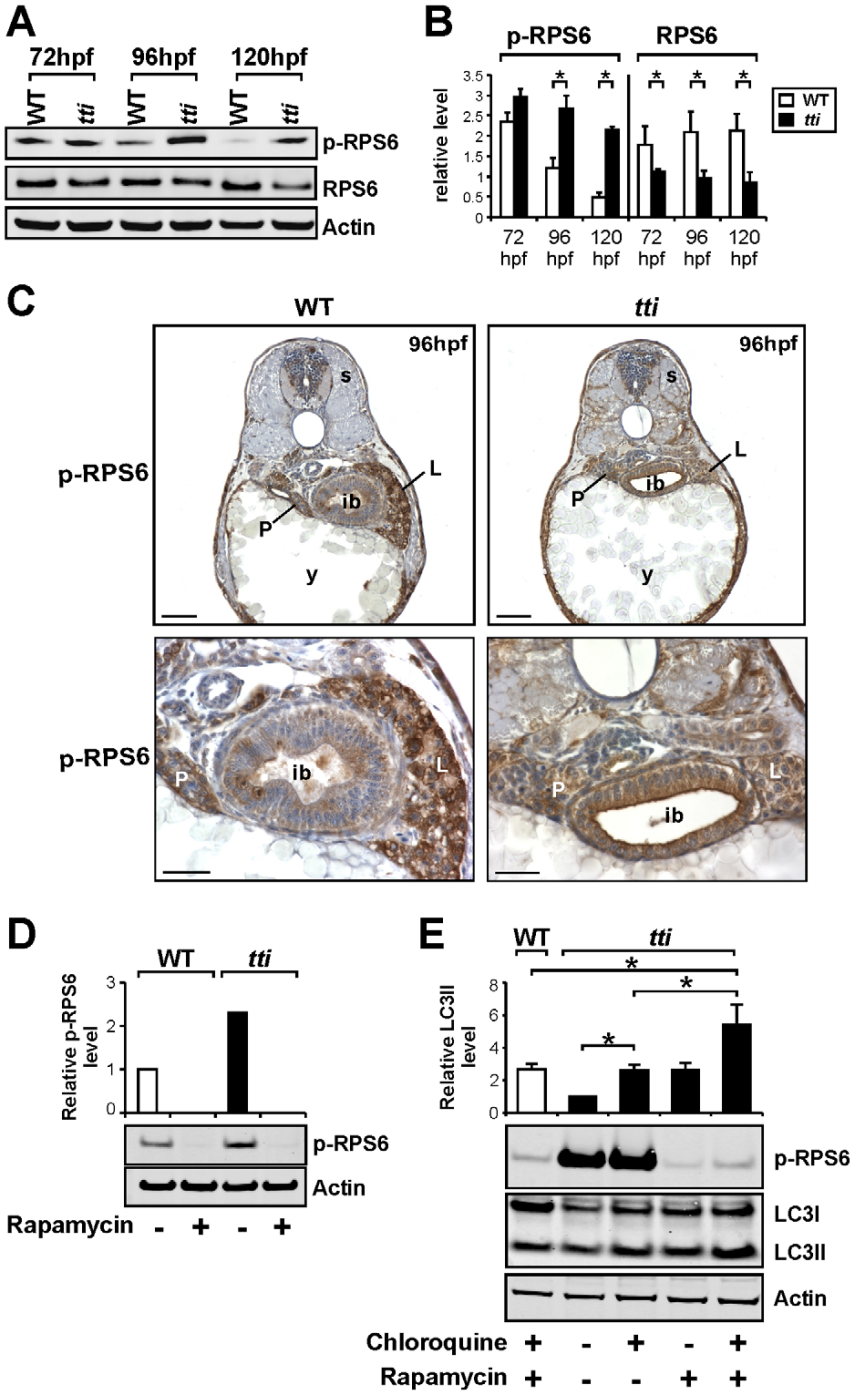
*tti^s450^* larvae exhibit elevated levels of Torc1 activity. (A) Western blot analysis of RPS6, p-RPS6 and Actin (loading control) in whole cell lysates of WT and *tti^s450^* larvae between 72–120 hpf. (B) Graphical representation of the data shown in A combined with two additional experiments (each bar represents the mean +/− SD, *p<0.05). *tti^s450^* larvae exhibit increased levels of p-RPS6 at 96–120 hpf and decreased levels of total RPS6 between 72–120 hpf compared to WT siblings. (C) Immunohistochemical analysis of transverse sections of *tti^s450^* and WT larvae at 96 hpf reveals robust p-RPS6 expression in the digestive organs. Scale bars = 50 µM. (D) The persistent expression of p-RPS6 expression in *tti^s450^* larvae at 96 hpf compared to WT is due entirely to up-regulated Torc1 activity as shown by the disappearance of the p-RPS6 signal when larvae are pre-treated with rapamycin. (E) Inhibiting the Tor pathway in *tti^s450^* larvae with rapamycin in the presence of chloroquine reduces p-RPS6 expression and at the same time increases autophagic flux as shown by the increase in LC3II level. In the graphical representation of the data, each bar represents the mean +/− SD (n = 3), *p<0.05.

To explore the relationship between the Tor pathway and autophagy in *tti^s450^* larvae, we analysed the levels of phosphorylated RPS6 (p-RPS6), a downstream target of Torc1 activity. Using Western blot analysis, we found that p-RPS6 levels decrease markedly in WT larvae between 72–120 hpf as previously reported [37] (Figure 7A, 7B). Somewhat surprisingly, p-RPS6 levels persist in *tti^s450^* larvae until 120 hpf, when they are 4-fold higher than in WT siblings (Figure 7A, 7B). We also noticed that the overall level of RPS6 protein is less in *tti^s450^* larvae compared to WT, perhaps reflecting the fact that RPS6 is a structural component of the 40S subunits, which are fewer in *tti^s450^* larvae. Using immunocytochemistry we examined p-RPS6 expression in histological sections of WT and *tti^s450^* larvae. At 96 hpf, we observed robust p-RPS6 expression in the intestinal epithelium and liver of WT and *tti^s450^* larvae (Figure 7C). The high p-RPS6 levels in the *tti^s450^* intestinal epithelium raise the possibility that elevated p-RPS6 stimulates autophagy directly in *tti^s450^* larvae, as this occurrence has been recognised previously, including in the *Drosophila* fat body during starvation [38], [39]. To test this, we blocked p-RPS6 accumulation using rapamycin. We found that prior exposure to rapamycin for 14 h eliminated the p-RPS6 signal in both WT and *tti^s450^* larvae at 96 hpf (Figure 7D), thereby unequivocally linking the persistent and elevated p-RPS6 signal in *tti^s450^* larvae to Torc1 activity. Moreover, rapamycin treatment of *tti^s450^* larvae in the presence and absence of chloroquine results in elevated levels of LC3II (Figure 7E) and LC3II-containing autophagosome formation (Figure 5F, 5G). These augmented levels of autophagy, achieved through rapamycin blockade of RPS6 phosphorylation, exclude the possibility that elevated p-RPS6 is responsible for the induction of autophagy in *tti^s450^* larvae. Indeed, these data suggest that autophagy induction in *tti^s450^* larvae is independent of the level of activation of the Tor pathway and the levels of p-RPS6.

We corroborated this finding with a genetic approach by crossing *tti^s450^* onto the *tsc2^vu242/vu242^* background [40]. Tsc2 is a negative regulator of Torc1 and *tsc2^vu242/vu242^* zebrafish larvae exhibit a variety of defects including an enlarged liver at 7 dpf [40], consistent with Tor playing a positive role in digestive organ growth. The development of the *tti^s450^* phenotype, including the induction of autophagy, is not perturbed on the *tsc2^vu242/vu242^* background (Figure S8A–S8F). Interestingly, *tti^s450^* larvae at 96 hpf contain higher levels of pRPS6 than *tsc2^vu242/vu242^* larvae (Figure S8E, S8F) and the levels of p-RPS6 are higher still in compound *tti^s450^*;*tsc2^vu242/vu242^* mutants (Figure S8E, S8F). In conclusion, these data show that impaired ribosome biogenesis induces autophagy in *tti^s450^* larvae through a mechanism that does not require inhibition of the Tor pathway and is independent of p-RPS6 levels.

### Autophagy induction in *tti^s450^* larvae is independent of Tp53

**Figure 8:**
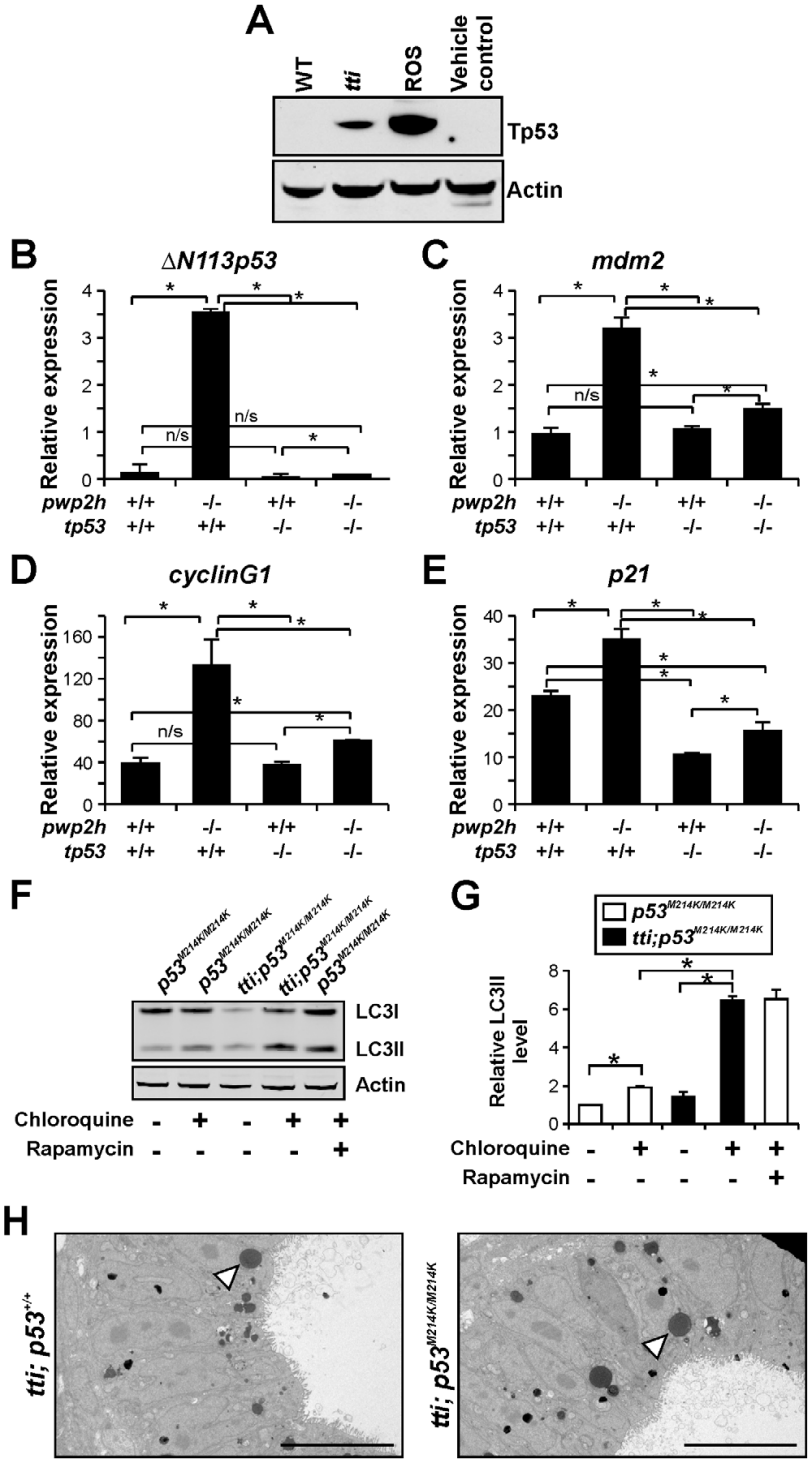
Autophagy in *tti^s450^* larvae is not due to Tp53 activation. (A) Western blot analysis of Tp53 protein in whole cell lysates of WT *(lane 1)* and *tti^s450^ (lane 2)* larvae at 96 hpf reveals up-regulation of Tp53 expression in *tti^s450^*. Larvae treated with roscovotine (ROS; *lane 3*) to induce Tp53 protein expression or untreated larvae (*lane 4*) are positive and negative controls, respectively. The Actin signal provides a loading control. (B–E) Relative expression of *ΔN113p53* (B), *mdm2* (C), *cyclinG1* (D) and *p21* (E) mRNAs in WT, *tti^s450^ (pwp2h^−/−^)*, *tp53^M214K/M214K^* (*tp53^−/−^*) and *tti^s450^;tp53^M214K/M214K^* (*pwp2h^−/−^;tp53^−/−^*) larvae at 96 hpf (n = 3) demonstrates that the expression of Tp53 target genes is increased in *tti^s450^* compared to WT larvae (compare first 2 bars in all graphs). The Tp53 response is diminished on the *tp53^M214K/M214K^* background, as expected (compare 2^nd^ and 4^th^ bars). Data were normalised by reference to Elongation factor alpha (Elf-α) expression. (F) Western blot analysis of LC3 in whole cell lysates of *tp53*-mutant (*tp53^M214K/M214K^*) and *tti^s450^;tp53^M214K/M214K^* larvae at 96 hpf. The elevated autophagic flux in *tti^s450^* larvae due to ribosomal stress is not diminished on a *tp53*-mutant background. (G) Graphical representation of the data shown in F and two additional experiments. Bars represent the mean +/− SD (n = 3), *p<0.05. (H) Transmission electron micrographs of IECs of *tti^s450^;tp53^M214K/M214K^* larvae at 120 hpf (*right panel*) reveal electron dense vesicles, resembling autolysosomes (*white arrowhead*), in comparable numbers to those found in *tti^s450^* larvae with WT Tp53 expression (*left panel*).

Defects in 18S and 28S rRNA processing have been shown to activate Tp53 [41], which in turn can stimulate autophagy [42]. While WT larvae contained negligible levels of Tp53 protein at 96 hpf, *tti^s450^* larvae display readily detectable levels of Tp53 protein at this time-point (Figure 8A) and increased transcription of Tp53 target genes, including *ΔN113p53*, *p21*, *cyclinG1* and *mdm2* (Figure 8B–8E). To determine whether Tp53 plays a role in the induction of autophagy in *tti^s450^*, we generated *tti^s450^* larvae expressing a mutant form of Tp53 (Tp53^M214K^) with negligible DNA-binding activity [43]. While this mutation severely diminished the elevated *ΔN113p53*, *p21*, *cyclinG1* and *mdm2* expression levels in *tti^s450^* larvae at 96 hpf as expected (Figure 8B–8E), the level of LC3II in compound *tti^s450^*;*tp53^M214K/M214K^* mutants in the presence of chloroquine was significantly higher than in *tp53^M214K/M214K^* mutants (Figure 8F–8H). In addition, ultrastructural analysis revealed similar numbers of autolysosomes in *tti^s450^* mutants at 120 hpf, independent of whether they were on the *tp53^M214K/M214K^* background or not (Figure 8H). Therefore the induction of autophagy in response to Pwp2h depletion proceeds unabated in *tti^s450^* larvae that are devoid of functional Tp53 protein.

## Discussion

This study shows, in the context of an intact vertebrate organism, that Pwp2h is critical for the production of mature 18S rRNA, an integral component of the 40S ribosomal subunit. In zebrafish, as in yeast, Pwp2h depletion results in reduced levels of the immediate precursor to mature 18S rRNA and a concomitant decrease in the production of mature 18S rRNA and assembly of 40S ribosomal subunits. Thus the role of Pwp2h in the 90S pre-ribosomal particle or small subunit processome is conserved from yeast to vertebrates.

In our *pwp2h*-deficient model, *titania* (*tti^s450^*), the growth of the endodermal organs, eyes, brain and craniofacial structures is severely arrested and autophagy is markedly up-regulated. To the best of our knowledge, this is the first time that a link between impaired ribosome biogenesis and autophagy has been demonstrated. We further show that elevated rates of autophagy support the survival of intestinal epithelial cells and increase the lifespan of *tti^s450^* larvae, thereby demonstrating that autophagy is a survival mechanism invoked in response to ribosomal stress. In our zebrafish model, autophagy induction does not depend on inhibition of the Tor pathway or activation of Tp53.

The death of *tti^s450^* larvae at 8–9 dpf demonstrates that *pwp2h* encodes a protein that is indispensable for life. However, the development of *tti^s450^* larvae until 72 hpf is supported by the deposition of maternal, wild-type *pwp2h* mRNA (and/or protein) into oocytes by their heterozygous mother. At 72 hpf, the tissues in which *pwp2h* is most highly expressed are the intestinal epithelium, pharyngeal arches, liver, dorsal midbrain, cerebellum, dorsal hindbrain, retinal epithelium and pancreas. These tissues are also the most rapidly proliferating tissues in WT larvae at 72 hpf [28] and the most severely affected tissues in *tti^s450^* larvae. Thus the tissue-specific phenotype of *tti^s450^* larvae may be explained by maternally-derived WT *pwp2h* mRNA being exhausted first in developing organs containing highly proliferative cells.

In WT zebrafish larvae there is a transient spike in Torc1 activity (as measured by p-RPS6) at around 72 hpf that is coincident with the activation of anabolic pathways required for cell growth and proliferation during the endoderm to intestine transition [37]. Torc1 is thought to play a role in developing organisms as an organ size checkpoint, potentiating growth signals that promote the rapid expansion of organs until they reach a genetically programmed cell size [44]. Therefore the persistent and robust activity of Torc1 we observe in the intestinal epithelium and liver of *tti^s450^* larvae at 96 hpf may be a consequence of these organs being markedly smaller than their WT counterparts at this stage.

The gross phenotype of *tti^s450^* is highly reminiscent of another zebrafish mutant, *nil per os (npo)*, in which the morphogenesis of the intestinal epithelium is also arrested. In *npo* the failure of the primitive gut endoderm to transform into a monolayer of polarized and differentiated epithelium is caused by a mutation in *rbm19*, a gene encoding a protein with six RNA recognition motifs that is also thought to play a role in ribosome biogenesis [45]. The same authors showed that essentially the same hypoplastic intestinal phenotype was recapitulated by exposure of WT zebrafish larvae to the Torc1 inhibitor, rapamycin [46], which presumably stimulated autophagy. It would be interesting to determine whether the growth arrest of the digestive organs in the *npo* mutant is also accompanied by autophagy.

The degree of activation of the Tor pathway is thought to be one of the major factors governing autophagy. However, Tor inhibition is not the mechanism responsible for autophagy in *tti^s450^* larvae and recent work suggests that autophagy regulation is a very complex process involving the integration of signals from many diverse signalling pathways [47]. Indeed, proteomic analysis of binding partners of components of the autophagy machinery suggests that several hundred molecules participate in the regulation of the human autophagy network [48]. While much recent attention has been focused on the direct phosphorylation of Ulk1/Atg1 by AMPK, acting either cooperatively or independently of Tor to exert autophagy control [19]–[21], there are many reports of other kinases capable of controlling autophagy by a variety of Tor-independent mechanisms [49]–[51]. The dissociation of the key BH3 domain-containing autophagy protein, Beclin 1 (mammalian orthologue of yeast Atg6) from its inhibitors Bcl2 and Bcl-XL as a result of phosphorylation of one or other components is also a critical determinant in the induction of autophagy [52]. In the case of *tti^s450^* larvae, it is plausible that autophagy induction may involve a targeted pathway, selective for ribosomes [11], which by analogy with mitophagy [53], is invoked to digest damaged cargo such as non-functional organelles.

Somewhat surprisingly, we also ruled out involvement of Tp53 in the induction of autophagy in *tti^s450^* larvae, even though Tp53 protein is active in *tti^s450^* larvae at 96 hpf. However, we believe the increased expression of Tp53 target genes such as *p21* and *cyclinG1* may be responsible, at least in part, for the reduction in the number of cells in the S phase of the cell cycle we observed at this time-point. To explain this, we surmise that as ribosome biogenesis is progressively impaired, the *tti^s450^* larvae mount a two-stage response to Pwp2h depletion. Initially, the cells undergo a Tp53-mediated cell cycle arrest. However, as the synthesis of new proteins, including Tp53 and its targets, is progressively impaired, the cells invoke autophagy to prolong their survival.

The notion of the existence of a second type of programmed cell death, distinct from apoptosis, which emanates from catastrophic levels of autophagy, is a hotly debated topic [54]. Using TEM, we did not see any evidence of cell death in the IECs of *tti^s450^* larvae, even at 7–8 dpf just before the larvae die, affirming that the levels of autophagy induced in the IECs of *tti^s450^* larvae prolong cell survival rather than trigger cell death. We proved this by disrupting the formation of the early autophagosome by inhibiting the translation of *atg5* mRNA. This resulted in the death of IECs in *tti^s450^* larvae and a markedly reduced lifespan.

As mentioned previously, *tti^s450^* larvae exhibit impaired development of the craniofacial cartilages, exocrine pancreas and brain, tissues that are often clinically abnormal in patients with certain human ribosomopathies, including Diamond Blackfan anaemia and Schwachman Diamond syndrome [4]. Recently, two new zebrafish models of dyskeratosis congenita (DC) based on mutations in components of the H/ACA RNP complex were described [55], [56]. Like *tti^s450^*, these mutants display impaired production of 18S rRNA and induction of Tp53 target genes, consistent with previous studies demonstrating that defects in ribosome biogenesis induce Tp53 activation and cell cycle arrest [41]. Moreover, hematopoietic stem cells in these mutants were depleted via a Tp53-dependent mechanism, providing a plausible explanation for why DC patients are susceptible to bone marrow failure [55], [56]. In one of these mutants, the gut and craniofacial structures were also reported to be underdeveloped and, as observed in *tti^s450^*, these defects persisted on a Tp53 mutant background [55]. We speculate that the p53-independent features of this model of DC may be caused by elevated rates of autophagy. If so, and these findings are confirmed in human DC, it will be important to determine whether elevated autophagic activity contributes to prolonged cell survival prior to considering clinical interventions to limit this process.

There is currently a great deal of interest in the development of novel therapeutics that target the cancerous translation apparatus through the combined inhibition of ribosome biogenesis, translation initiation and translation elongation [5]. To avoid inadvertently prolonging cancer cell survival, these approaches could benefit from a detailed understanding of the mechanisms and cellular contexts that induce autophagy in response to ribosomal stress. While such insights may be forthcoming from studies performed on cell lines, it is likely that complementary experiments carried out in the context of an entire vertebrate organism, such as the zebrafish model introduced here, may also be fruitful.

## Materials and Methods

### Ethics statement

All experimental procedures on zebrafish embryos and larvae were approved by the Ludwig Institute for Cancer Research/Department of Surgery - Royal Melbourne Hospital Animal Ethics Committee.

### Zebrafish strains and embryo collection

Zebrafish embryos were obtained from pair-wise matings of heterozygous *tti^s450^*, *setebos^s450^* and *caliban^s846^* zebrafish on the *Tg(XlEef1a1:GFP)^s854^* (*gutGFP*) background and from *tti* ^s450^ heterozygotes carrying two mutant alleles of *Tp53* (*tti^s450^*;*Tp53* ^M214K/M214K^) [43] and raised at 28.5°C. *tti* ^s927^ was propagated on the *Tg*(*ins*:*dsRed*)*^m1081^*;*Tg*(*fabp10*:*dsRed*;*ela3l*:*GFP*)*^gz12^* (2-CLIP) background [31]. The *Tp53* ^M214K/M214K^ line (gift of Thomas Look and David Lane) and *tsc2^vu24^* line were obtained through TILLING [40], [43]. The *tsc2* and *pwp2h* loci in zebrafish are both on chromosome 1 so in order to generate sufficient *tti^s450^;tsc2^vu24^* compound mutants for analysis, we identified and in-crossed recombinants harbouring the two mutations in a *cis* configuration. To prevent melanization and maintain transparency, embryos were treated with 0.003% 1-phenyl-2-thiourea (PTU; Sigma Aldrich) in embryo medium. Imaging of live larvae was carried out using a LeicaM2 FLIII microscope after anaesthetizing with 200 mg/L benzocaine (Sigma-Aldrich, St. Louis, MO) in embryo medium. All images were imported into CorelDRAWX4 (Corel Corporation, Ottawa, Ontario, Canada). Image manipulation was limited to levels, hue and saturation adjustments.

### Histology and whole-mount *in situ* hybridisation

Histology was performed as described [27]. Mucins and other carbohydrates secreted by intestinal goblet cells were stained using alcian blue-periodic acid-Schiff reagent [27]. For WISH, larvae were processed as described [57], [58] To generate *pwp2h* riboprobes a cDNA template was amplified by RT-PCR. For primer sequences see Text S1. These were then transcribed using the digoxigenin DNA Labelling Kit (Roche Diagnostics) according to the manufacturer's instructions. Hybridized riboprobes were detected using an anti-DIG antibody conjugated to alkaline phosphatase according to the manufacturer's instructions (Roche Diagnostics). Larvae were imaged on a Nikon SMZ 1500 microscope.

### Fluorescence-activated cell sorting (FACS)

100–200 WT and *tti* ^s450^ larvae were rinsed in PBST (PBS containing 0.5% Tween 20) three times prior to incubating in 1 mL Hank's Balanced Salt Solution containing 0.25% trypsin, 0.1% EDTA, 40 µg/mL Proteinase K and 10 µg/mL collagenase for 30 min at 37°C. Larvae were then homogenised in 7 mL PBS containing 5% FBS. The cell suspension was strained through a 40 µM nylon cell strainer (BD Falcon) and spun at 2000 rpm for 10 min at 4°C. The pellet was washed twice with cold PBS/5% FBS and resuspended in 500 µl PBS. Ice-cold methanol (900 µl) was added to the pellet and cells were left on ice for 1 h prior to centrifugation as above. The pellet was resuspended in 0.5 mL PBS containing 40 µg/mL propidium iodide and 0.5 mg/mL RNaseA for 30–60 min at room temperature (RT). GFP positive cells were sorted on a FACSCalibur™ Optics instrument (Benton Dickinson) and analysis was performed using the ModFit LT program.

### Detection of cells in the S-Phase of the cell cycle and cell height determination

To identify cells in the S-phase of the cell cycle, the incorporation of bromodeoxyuridine (BrdU) by live larvae was analysed as described [27]. To measure cell height, images of sagittal histological sections were captured on a Nikon Eclipse 80i microscope and then analysed using MetaMorph Microscopy Automation & Image Analysis Software.

### Genetic mapping and positional cloning of *tti^s450^*

For genetic mapping, *tti* ^s450^ heterozygotes on the gutGFP background were crossed onto the polymorphic WIK strain. Mutant larvae were identified by craniofacial and intestinal defects visible at 96 hpf under brightfield and fluorescence illumination. Subsequent mapping was performed as described [28].

### Sequence alignment and domain determination

Protein sequence alignment of Pwp2h from zebrafish, yeast, mouse and human was performed using the clustalW2 program with default parameters. WD domains were identified using the Simple Modular Architecture Research Tool (SMART) software.

### Genotyping

A novel *EcoN1* restriction enzyme site created by the *tti^s450^* mutation produced a restriction fragment length polymorphism (RFLP) that was exploited for genotyping. Primers were used to amplify a 653-base pair (bp) fragment spanning exons 9 to 11 containing the *tti^s450^* mutation. For primer sequences see Text S1.

### RNA preparation and Northern blot analysis

Total cellular RNA was prepared from WT and *tti^s450^* larvae (120 hpf) by homogenizing 20–50 larvae in Solution D (4.2 M guanidinium thiocyanate, 25 mM NaCitrate, 30% Sarkosyl BDH NL30) as described [59]. Northern blot analysis was conducted on 2 µg samples using α-^32^P-labelled probes designed to hybridize to zebrafish 5′ETS, ITS1 and ITS2 sequences, which were PCR-amplified from genomic DNA using previously described primers [60]. Radioactive signals were detected using a Phosphorimager and Storm 820 scanner (Amersham Biosciences) and analysed using ImageQuant TL software.

### Analysis of 18S and 28S rRNA levels

Solutions of total RNA extracted from WT and *tti^s450^* larvae were analysed on an Agilent 2100 E-Bioanalyser according to the manufacturer's instructions.

### Polysome fractionation

50–100 WT and *tti^s450^* larvae at 96 hpf were resuspended in cold lysis buffer (50 mM Tris-HCl pH 7.4, 150 mM KCl, 2.5 mM MgCL_2_, 1% Triton X-100, 0.5% sodium deoxycholate, 3 mM DTT) containing 120 U/mL RNase inhibitor (Invitrogen) and Complete Protease Inhibitor Cocktail (Roche) and sheared through a 23G needle. Lysates were incubated on ice for 30 min and centrifuged (12,000 rpm, 20 min at 4°C) to pellet nuclei and cellular debris. Cytoplasmic extract (2 mg) was loaded onto a continuous low salt (80 mM NaCl) 3.1–30.1% (w/v) sucrose gradient (14 mL) [61] generated using an ISCO gradient maker. Samples were separated by centrifugation using a SW41 rotor at 40000 rpm for 4 h at 4°C, and fractionated (1 mL) using a Foxy Jr fraction collector. Absorbance at 260 nM was determined with an ISCO UA-6 absorbance detector. In each case, quantitation of 40S, 60S, and 80S was performed by measuring the area under the relevant peak using Metamorph Image Analysis Software.

### Transmission electron microscopy (TEM)

For TEM, larvae were fixed in 2.5% glutaraldehyde, 2% paraformaldehyde (Electron Microscopy Sciences, Hatfield, PA) in PBS for 2 h at R.T, rinsed in 0.08 M Sorensen's Phosphate buffer pH 7.4 and then stored in 0.08 M Sorensen's buffer with 5% sucrose. Post-fixation was with 2% osmium tetroxide in PBS followed by dehydration through a graded series of alcohols, 2 acetone rinses and embedding in Spurrs resin [62]. Sections approximately 80 nm thick were cut with a diamond knife (Diatome, Switzerland) on a Ultracut-S ultramicrotome (Leica, Mannheim, Germany) and contrasted with uranyl acetate and lead citrate. Images were captured with a Megaview II cooled CCD camera (Soft Imaging Solutions, Olympus, Australia) in a JEOL 1011 TEM. Transverse sections were obtained through the anterior intestinal region known as the intestinal bulb.

### Immunocytochemistry

For transverse sections, embryos were fixed in 2% paraformaldehyde overnight at 4°C, embedded vertically in 4% low melting temperature agarose (Cambrex BioScience, East Rutherford, NJ) in disposable cryomolds (Sakura Finetek, Torrance, CA), and sectioned at 200 µm intervals using a Leica (Solms, Germany) VT1000S vibrating microtome. Floating sections were transferred to the wells of a 24-well plate containing PBD (PBS containing 0.1% Tween-20 and 0.5% Triton-X) and then replaced with antibody blocking solution (PBD containing 1% (w/v) BSA and 1% (v/v) FCS) for 2 h at RT. The blocking solution was removed and the sections incubated with LC3B primary antibody diluted to 1∶500 in PBD containing 0.2% (w/v) BSA at 4°C overnight. The sections were rinsed three times in PBST (PBS containing 0.1% Tween-20) for 20 min at RT, followed by antibody blocking solution for 2 h at RT. The sections were then incubated overnight at 4°C in PBD containing 0.2% (w/v) BSA, Alexa Fluor 488 (1∶500), rhodamine-phalloidin (1∶150; Biotium, Hayward, CA) and 5 µg/mL Hoechst33342 (Sigma Aldrich). Sections were rinsed three times in PBST for 20 min at RT prior to imaging on an Olympus FV1000 scanning confocal microscope. Enumeration of LC3 puncta was performed using Metamorph. Details of antibodies and stains are available in Text S1.

### Western blot analysis

Larvae were lysed (2 µL per embryo) in cold RIPA cell lysis buffer (50 mM Tris-HCl pH 7.4, 150 mM NaCl, 2 mM EDTA, 1% NP-40, 0.1% SDS) containing Complete Protease Inhibitor Cocktail (Roche) and sheared through a 23G needle. Lysates were incubated on ice for 30 min and then centrifuged for 20 min at 13,000 rpm at 4°C to pellet nuclei and cellular debris. Samples containing 40–80 µg of protein were heated to 95°C for 5 min with 5 X Protein Loading Dye (0.03 M Tris-HCl, pH 6.8, 13.8% glycerol, 1% SDS, 0.05% bromophenol blue, 2.7% β-mercaptoethanol) and loaded onto a 12% polyacrylamide gel. The proteins were transferred to PVDF membranes using an iBlot Gel Transfer Device (Invitrogen) according to the manufacturer's instructions. For RPS6, p-RPS6, LC3 and Actin, subsequent blocking, antibody incubation and membrane exposure were performed using the Odyssey system (LI-COR Biosciences). For Tp53, blocking and antibody incubation were performed in PBST/5% skim milk powder and membranes developed using the SuperSignal West Femto Chemilluminescent Substrate (Thermo Scientific). Signals were quantitated by densitometry and expressed as relative levels by reference to the level in untreated WT larvae, which was set at 1. Details of antibodies are provided in Text S1.

### Expression of mCherry-LC3 fusion protein

DNA encoding the fluorophore mCherry fused to the N terminus of LC3 was PCR amplified and transcribed into mRNA using the mMessage mMachine SP6 kit (Ambion Life Technologies, Mulgrave, Australia). For primer sequences see Text S1. mRNA (400 pg) was injected into the yolk of 1–4 cell stage embryos and exposed to 2.5 µM chloroquine (Fluka Sigma-Aldrich, Sydney, Australia) in embryo medium for 14 h at various time-points during development prior to mounting in 1.5% low melting point agarose for imaging with an Olympus FV1000 scanning confocal microscope.

### Drug treatment

Live WT, *tti^s450^*, *set^s453^ clbn^s846^* larvae were exposed to 2.5 µM chloroquine and/or 10 µM rapamycin in embryo medium at 28°C. Larvae were collected 14 h later for protein extraction and Western blot analysis of LC3II levels as described above.

### Knockdown of Pwp2h and Atg5 protein expression

Antisense morpholino oligonucleotides (MOs) targeted to the initiation of translation codons of *pwp2h* or *atg5* mRNA were injected into the yolk of 1–4 cell stage WT or *tti^s450^* embryos. 2 nL of MO at a concentration of 120×10^−15^ mol (total = 1 ng) and 180×10^−14^ mol (total = 15 ng) were used to knockdown *atg5* and *pwp2h* mRNA translation, respectively. For MO sequences see Text S1.

### Quantitation of autophagosomes

Using immunocytochemical analysis, LC3II-containing autophagosomes were identified as puncta in thick transverse sections of *tti^s450^* larvae. Puncta in 20 cells in 3 independent sections were counted using Metamorph. For TEM sections, the numbers of autophagosome-like structures in 20 cells in 3 independent sections were counted manually.

### Quantitative reverse transcription polymerase chain [61] reaction (qRT–PCR)

cDNA was reverse transcribed from total RNA (1–2 µg) extracted from WT and *tti^s450^* larvae at 96 hpf using the Superscript III First Strand Synthesis System (Invitrogen) according to manufacturer's instructions. qRT-PCR was performed using the SensiMix SYBR Kit (Bioline) according to manufacturer's instructions. For primer sequences see Text S1.

### Statistical methods

Student's t-test was used to compare the means of two populations in Graphpad Prism 5.0. Error bars represent the mean +/− standard deviation (n≥3). A P value<0.05 was used to define statistical significance.

## Supporting Information

Figure S1 *tti^s450^* larvae contain fewer replicating IECs than WT larvae. (A) Sagittal sections of the intestine of WT and *tti^s450^* zebrafish larvae at 72 hpf showing cells that accumulated BrdU (black arrows) during a 30 min exposure to this thymidine analogue at 72 hpf. BrdU-positive nuclei (brown) indicate cells in the S-phase of the cell cycle. Scale bars = 50 µm. (B) Quantitation of BrdU-positive IECs in three independent sagittal sections of WT and *tti^s450^* larvae at 72 hpf reveals that *tti^s450^* larvae contain approximately 50% fewer S-phase IECs than WT. *p<0.05. Data are represented as mean +/− SD.
(TIF)

Figure S2 *pwp2h* is the mutated gene in *tti^s450^*. (A) Sequence of *pwp2h* in WT and *tti^s450^* cDNA reveals that *tti^s450^* larvae utilize a cryptic splice site in exon 10 due to a mutation in the splice acceptor site in intron 9. This results in an 11 bp deletion *(bracket)* which causes a frame-shift in the *pwp2h* coding sequence resulting in 13 aberrant amino acids and a premature stop codon in exon 10. (B, C) Upon microinjection into the yolk of 1–4 cell WT zebrafish embryos, a *pwp2h*-targeted MO (15 ng) produces a robust *tti^s450^* phenotype at 120 hpf (C). Vehicle-injected controls appear WT (B). (D–G) Non-complementation of 2 independent *pwph2* alleles confirms that *pwph2* is the mutated gene in *tti^s450^*. Heterozygous *tti^s450^* carriers were crossed with heterozygous carriers of *s927*, an independent *pwph2* allele identified in the 2-CLIP screen [30]. One quarter of the offspring are compound *tti^s450^*;*tti^s927^* mutants (E) and exhibit the *tti^s450^* phenotype (F) at 120 hpf including impaired development of the digestive organs, eye and craniofacial structures. Other panels show WT (D) and *tti^s927^* mutant (G) larvae at 120 hpf. These data indicate that both alleles correspond to the same genetic locus. e, eye; ib, intestinal bulb; sb, swim bladder; y, yolk. (H) The nucleotide sequence of *pwp2h* cDNA generated from *tti^s927^* larvae contains a T→A transversion (arrow). (I) The base change in *tti^s927^* results in a highly conserved branched amino acid (valine, *shaded blue*) being replaced by glutamic acid. Alignment was performed using ClustalW.
(TIF)

Figure S3 Alignment of human, mouse, zebrafish and yeast Pwp2h protein sequences. Zebrafish Pwp2h protein comprises 937 amino acids, compared with 919 in human and mouse and 923 in yeast. WD domains are highly conserved *(shaded in blue)*. The position of the amino acid change in *tti^s927^* larvae occurs at amino acid 113 in the 2^nd^ WD domain *(red box)*. The position where the frame-shift occurs in *tti^s450^* is indicated *(red arrow)* as is the position of the premature stop codon *(red star)*. Sequences used: human (*Homo sapiens*) NP_005040.2; mouse (*Mus musculus*) NP_083822.1; zebrafish *(Danio rerio)* NP_998212.1; yeast *(Saccharomyces cerevisiae)* NP_009984.1.
(TIF)

Figure S4 LC3II-containing autophagosomes are found in multiple tissues in *tti^s450^* larvae at 72 hpf and 120 hpf. (A–H) RNA encoding a mCherry-LC3 fusion protein was injected into the yolk of 1–4 cell zebrafish embryos derived from a pairwise mating of *tti^s450/+^* heterozygotes (on the gutGFP background) and allowed to develop until the indicated time-point in the presence of chloroquine for the final 14 h. Maximum intensity projection images of a z series of confocal sections through WT [A, A′ (boxed area in A), C, E, E′ (boxed area in E) and G] and *tti^s450^* larvae [B, B′ (boxed area in B), D, F, F′ (boxed area in F) and H] showing accumulated autophagosomes *(red puncta)* in the brain, eye and digestive organs (marked by GFP fluorescence in C, D) at 72 hpf (A–D) and 120 hpf (E–H). Scale bars = 50 µM. b, brain; e, eye; ib, intestinal bulb; f, fin; y, yolk; p, pancreas.
(TIF)

Figure S5 Up-regulated autophagy is not a shared feature of all zebrafish intestinal mutants. (A) Western blot analysis of LC3 in protein extracts of WT, *setebos (set^s453^)* and *caliban (clbn^s846^)* larvae. Actin was used as a loading control. (B) The levels of LC3II were quantitated by densitometric analysis of three independent Western blots. Chloroquine-treated *set^s453^* larvae at 96 hpf contain significantly higher LC3II levels compared to their chloroquine-treated WT siblings; meanwhile, LC3II levels are similar in chloroquine-treated *set^s453^* larvae and WT larvae treated with rapamycin and chloroquine. There are no significant differences between LC3II levels in *clbn^s846^* larvae and their WT siblings at 120 hpf, in the presence and absence of chloroquine. Data are represented as mean +/− SD (n = 3), *p<0.05. (C–H) Transmission electron micrographs of transverse sections of WT (C, E, G) and *clbn^s846^* larvae (D, F, H) through the intestinal bulb region at 120 hpf. There are negligible numbers of autophagosomes/autolysosomes in the IECs of WT and *clbn^s846^* larvae. Scale bars = 50 µm (C, D); 10 µm (E, F); 5 µm (G–H). ib, intestinal bulb; n, nucleus; m, mitochondria; mv, microvilli.
(TIF)

Figure S6 Absence of dead cells in the intestinal lumen of WT and *tti^s450^* larvae at 7 dpf. (A–F) Transmission electron micrographs of transverse sections of WT and *tti^s450^* larvae at 168 hpf (7 dpf). The number of conspicuous autophagosome-like structures in the IECs of *tti^s450^* larvae has diminished by 7 dpf and there are no dead cells in the lumen (D). Meanwhile, liver cells of *tti^s450^* larvae contain abundant autolysosome-like structures at this time-point (F, *white arrows*). Scale bars = 50 µm (A, B); 10 µm (C–F). ib, intestinal bulb; n, nucleus; m, mitochondria; mv, microvilli; l, liver; bd, bile duct; a, arteriole.
(TIF)

Figure S7 Disruption of autophagy in *tti^s450^* larvae results in severe oedema. Upon microinjection into the yolk of 1–4 cell WT and *tti^s450^* zebrafish embryos, an *atg5*-targeted MO (1 ng) produces severe oedema around the organs of *tti^s450^* larvae at 120 hpf (D), while WT larvae are unaffected (C). WT and *tti^s450^* larvae injected at the 1–4 cell stage with vehicle (A, B) are also unaffected.
(TIF)

Figure S8 Autophagic flux in *tti^s450^* larvae is not abrogated by Tor pathway activation. (A–D) Enhancing Torc1 activity by ablating Tsc2 activity in *tti^s450^* larvae does not change their gross morphology at 120 hpf. Compound mutants (*tti^s450^;Tsc2^vu242/vu242^*) (D) are essentially indistinguishable from *tti^s450^* larvae (C). Other panels show WT (A) and *Tsc2^vu242/vu242^* mutant (B) larvae. (E,F) Western blot analysis of p-RPS6 and LC3 demonstrates that *tti^s450^;Tsc2^vu242/vu242^* compound mutants at 96 hpf contain higher levels of p-RPS6 than *tti^s450^* mutants due to increased Tor activity, yet LC3II levels are comparable between the two genotypes (refer to right hand half of the Western blot, where the larvae were pre-treated with chloroquine). p-RPS6 and LC3II levels are not significantly different between WT and *tsc2^vu242/vu242^* larvae in the presence of chloroquine. Actin was used as a loading control. The levels of LC3II were quantitated by densitometric analysis of three independent Western blots. Data are represented as mean +/− SD, *p<0.05.
(TIF)

Text S1 Sequences of primers and morpholinos and additional antibody information.
(PDF)
