# Supplementary figures and images for: Autophagy Induction Is a Tor- and Tp53-Independent Cell Survival Response in a Zebrafish Model of Disrupted Ribosome Biogenesis
Source: PLoS Genet. 2013 Feb 7;9(2):e1003279. doi: 10.1371/journal.pgen.1003279 (PMC3567153; doi:10.1371/journal.pgen.1003279)

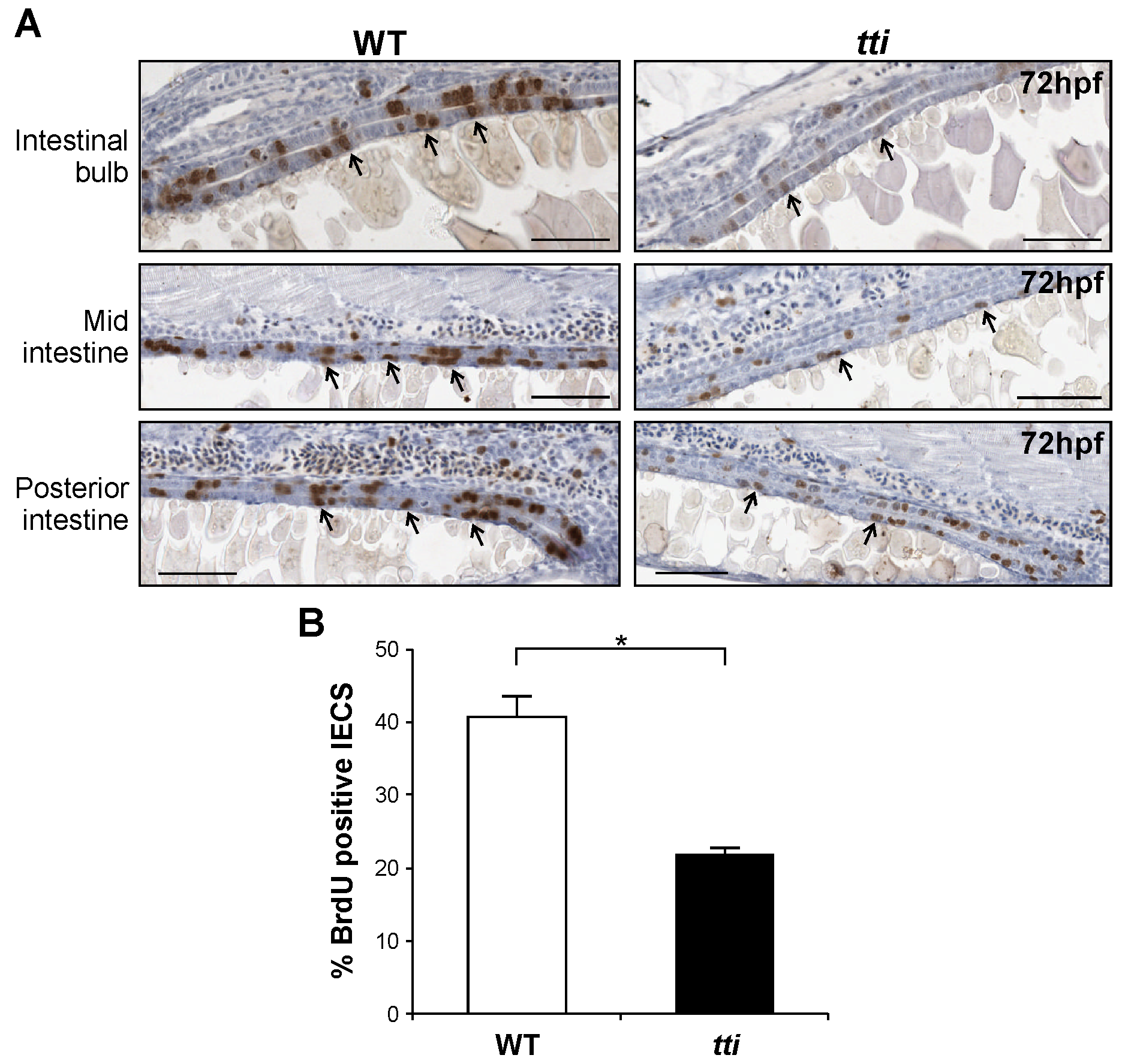

Supplement: Figure S1 — ttis450 larvae contain fewer replicating IECs than WT larvae. (A) Sagittal sections of the intestine of WT and ttis450 zebrafish larvae at 72 hpf showing cells that accumulated BrdU (black arrows) during a 30 min exposure to this thymidine analogue at 72 hpf. BrdU-positive nuclei (brown) indicate cells in the S-phase of the cell cycle. Scale bars = 50 µm. (B) Quantitation of BrdU-positive IECs in three independent sagittal sections of WT and ttis450 larvae at 72 hpf reveals that ttis450 larvae contain approximately 50% fewer S-phase IECs than WT. *p<0.05. Data are represented as mean +/− SD. (TIF) [file pgen.1003279.s001.tif]

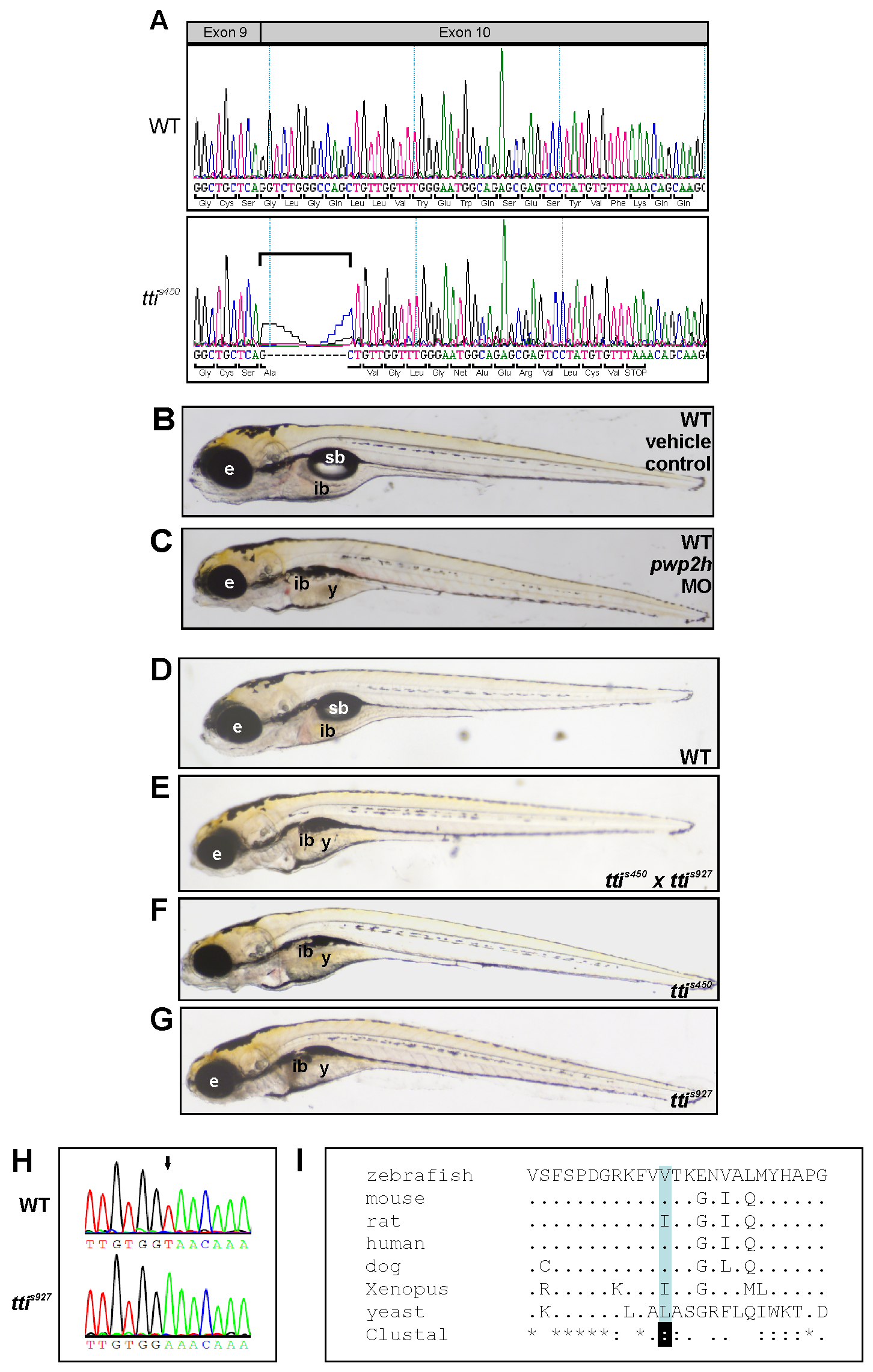

Supplement: Figure S2 — pwp2h is the mutated gene in ttis450. (A) Sequence of pwp2h in WT and ttis450 cDNA reveals that ttis450 larvae utilize a cryptic splice site in exon 10 due to a mutation in the splice acceptor site in intron 9. This results in an 11 bp deletion (bracket) which causes a frame-shift in the pwp2h coding sequence resulting in 13 aberrant amino acids and a premature stop codon in exon 10. (B, C) Upon microinjection into the yolk of 1–4 cell WT zebrafish embryos, a pwp2h-targeted MO (15 ng) produces a robust ttis450 phenotype at 120 hpf (C). Vehicle-injected controls appear WT (B). (D–G) Non-complementation of 2 independent pwph2 alleles confirms that pwph2 is the mutated gene in ttis450. Heterozygous ttis450 carriers were crossed with heterozygous carriers of s927, an independent pwph2 allele identified in the 2-CLIP screen [30]. One quarter of the offspring are compound ttis450;ttis927 mutants (E) and exhibit the ttis450 phenotype (F) at 120 hpf including impaired development of the digestive organs, eye and craniofacial structures. Other panels show WT (D) and ttis927 mutant (G) larvae at 120 hpf. These data indicate that both alleles correspond to the same genetic locus. e, eye; ib, intestinal bulb; sb, swim bladder; y, yolk. (H) The nucleotide sequence of pwp2h cDNA generated from ttis927 larvae contains a T→A transversion (arrow). (I) The base change in ttis927 results in a highly conserved branched amino acid (valine, shaded blue) being replaced by glutamic acid. Alignment was performed using ClustalW. (TIF) [file pgen.1003279.s002.tif]

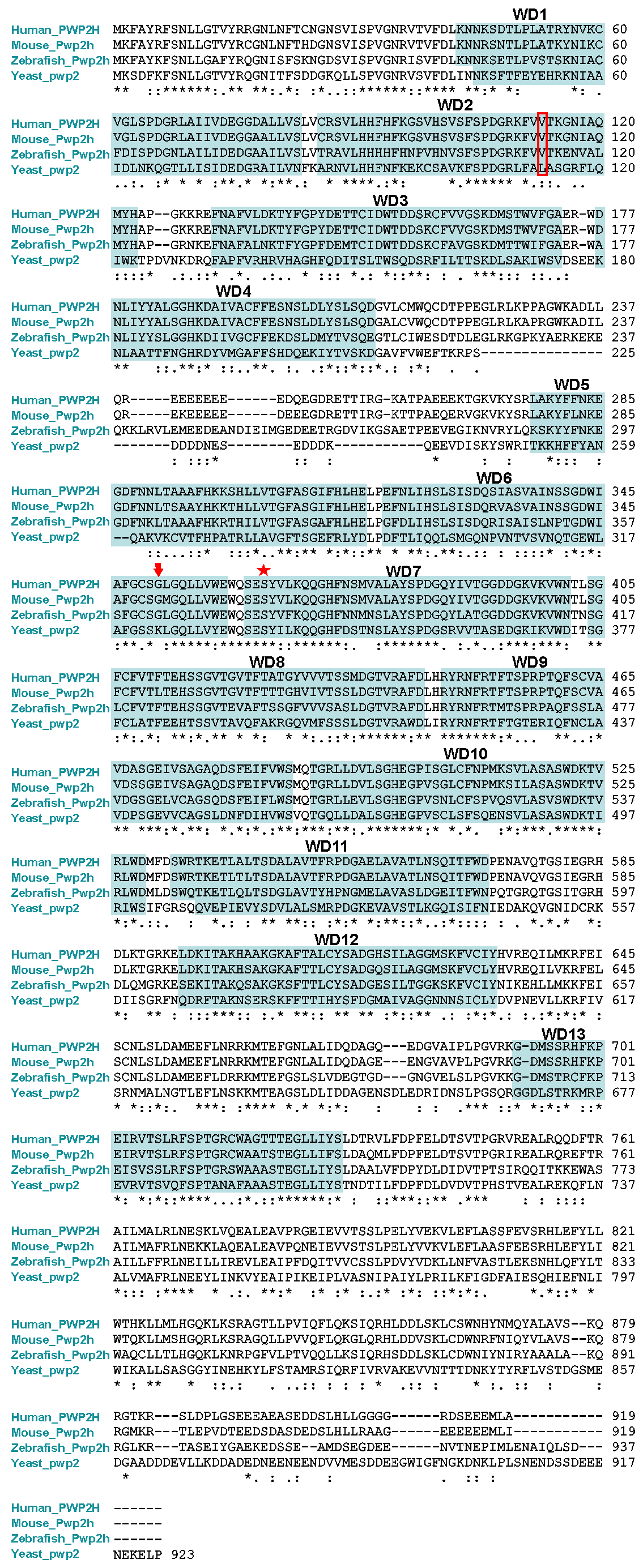

Supplement: Figure S3 — Alignment of human, mouse, zebrafish and yeast Pwp2h protein sequences. Zebrafish Pwp2h protein comprises 937 amino acids, compared with 919 in human and mouse and 923 in yeast. WD domains are highly conserved (shaded in blue). The position of the amino acid change in ttis927 larvae occurs at amino acid 113 in the 2nd WD domain (red box). The position where the frame-shift occurs in ttis450 is indicated (red arrow) as is the position of the premature stop codon (red star). Sequences used: human (Homo sapiens) NP_005040.2; mouse (Mus musculus) NP_083822.1; zebrafish (Danio rerio) NP_998212.1; yeast (Saccharomyces cerevisiae) NP_009984.1. (TIF) [file pgen.1003279.s003.tif]

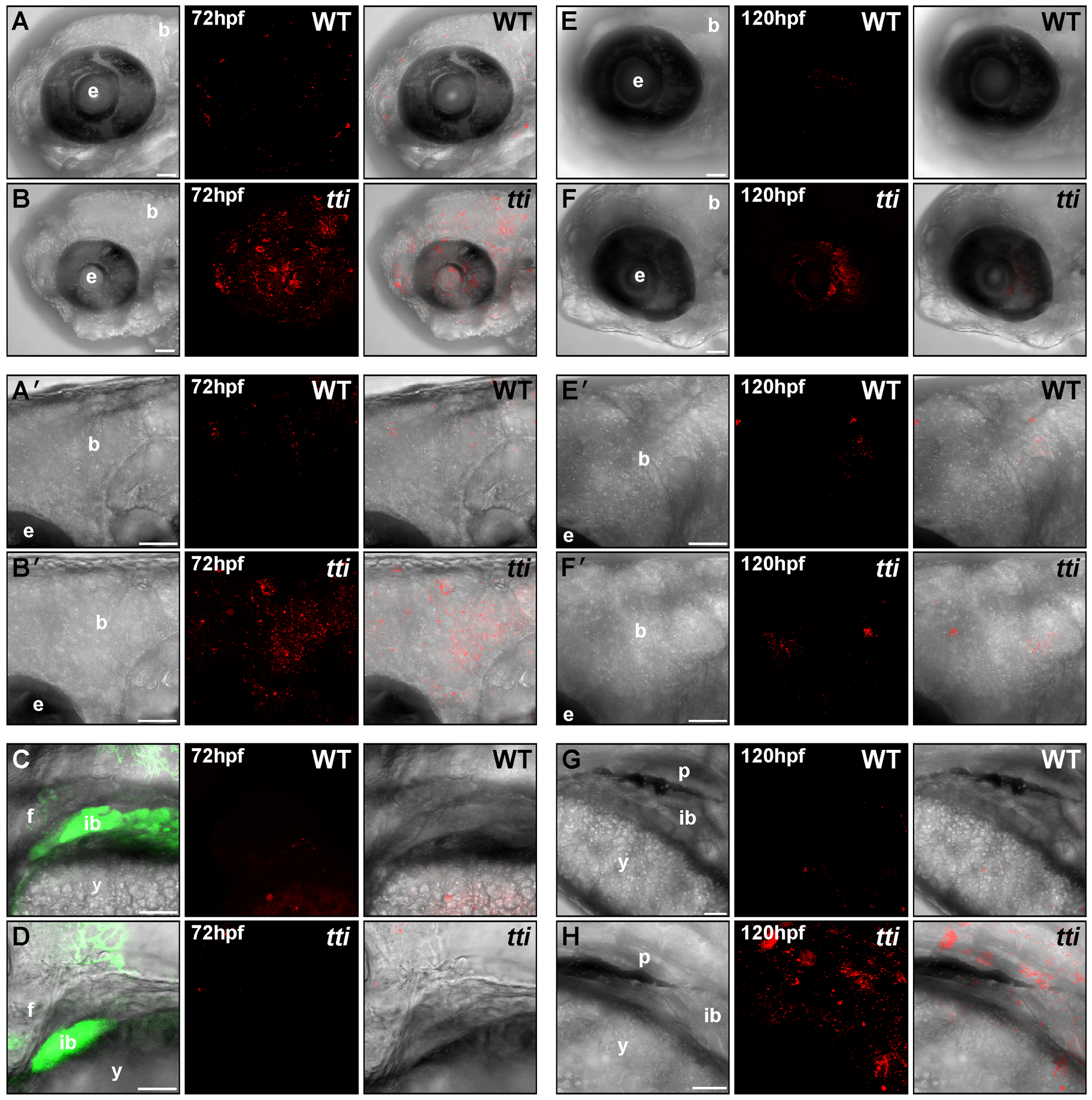

Supplement: Figure S4 — LC3II-containing autophagosomes are found in multiple tissues in ttis450 larvae at 72 hpf and 120 hpf. (A–H) RNA encoding a mCherry-LC3 fusion protein was injected into the yolk of 1–4 cell zebrafish embryos derived from a pairwise mating of ttis450/+ heterozygotes (on the gutGFP background) and allowed to develop until the indicated time-point in the presence of chloroquine for the final 14 h. Maximum intensity projection images of a z series of confocal sections through WT [A, A′ (boxed area in A), C, E, E′ (boxed area in E) and G] and ttis450 larvae [B, B′ (boxed area in B), D, F, F′ (boxed area in F) and H] showing accumulated autophagosomes (red puncta) in the brain, eye and digestive organs (marked by GFP fluorescence in C, D) at 72 hpf (A–D) and 120 hpf (E–H). Scale bars = 50 µM. b, brain; e, eye; ib, intestinal bulb; f, fin; y, yolk; p, pancreas. (TIF) [file pgen.1003279.s004.tif]

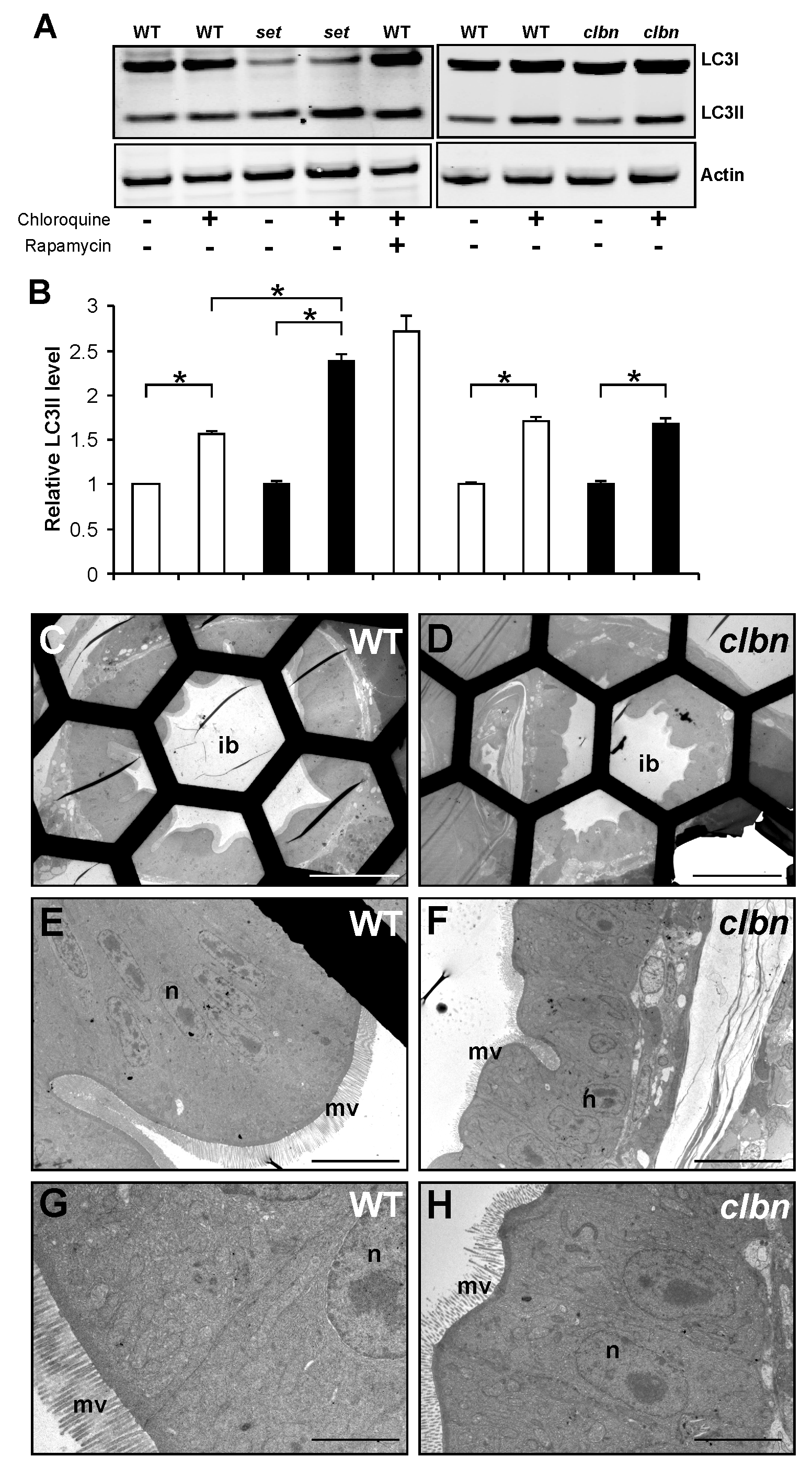

Supplement: Figure S5 — Up-regulated autophagy is not a shared feature of all zebrafish intestinal mutants. (A) Western blot analysis of LC3 in protein extracts of WT, setebos (sets453) and caliban (clbns846) larvae. Actin was used as a loading control. (B) The levels of LC3II were quantitated by densitometric analysis of three independent Western blots. Chloroquine-treated sets453 larvae at 96 hpf contain significantly higher LC3II levels compared to their chloroquine-treated WT siblings; meanwhile, LC3II levels are similar in chloroquine-treated sets453 larvae and WT larvae treated with rapamycin and chloroquine. There are no significant differences between LC3II levels in clbns846 larvae and their WT siblings at 120 hpf, in the presence and absence of chloroquine. Data are represented as mean +/− SD (n = 3), *p<0.05. (C–H) Transmission electron micrographs of transverse sections of WT (C, E, G) and clbns846 larvae (D, F, H) through the intestinal bulb region at 120 hpf. There are negligible numbers of autophagosomes/autolysosomes in the IECs of WT and clbns846 larvae. Scale bars = 50 µm (C, D); 10 µm (E, F); 5 µm (G–H). ib, intestinal bulb; n, nucleus; m, mitochondria; mv, microvilli. (TIF) [file pgen.1003279.s005.tif]

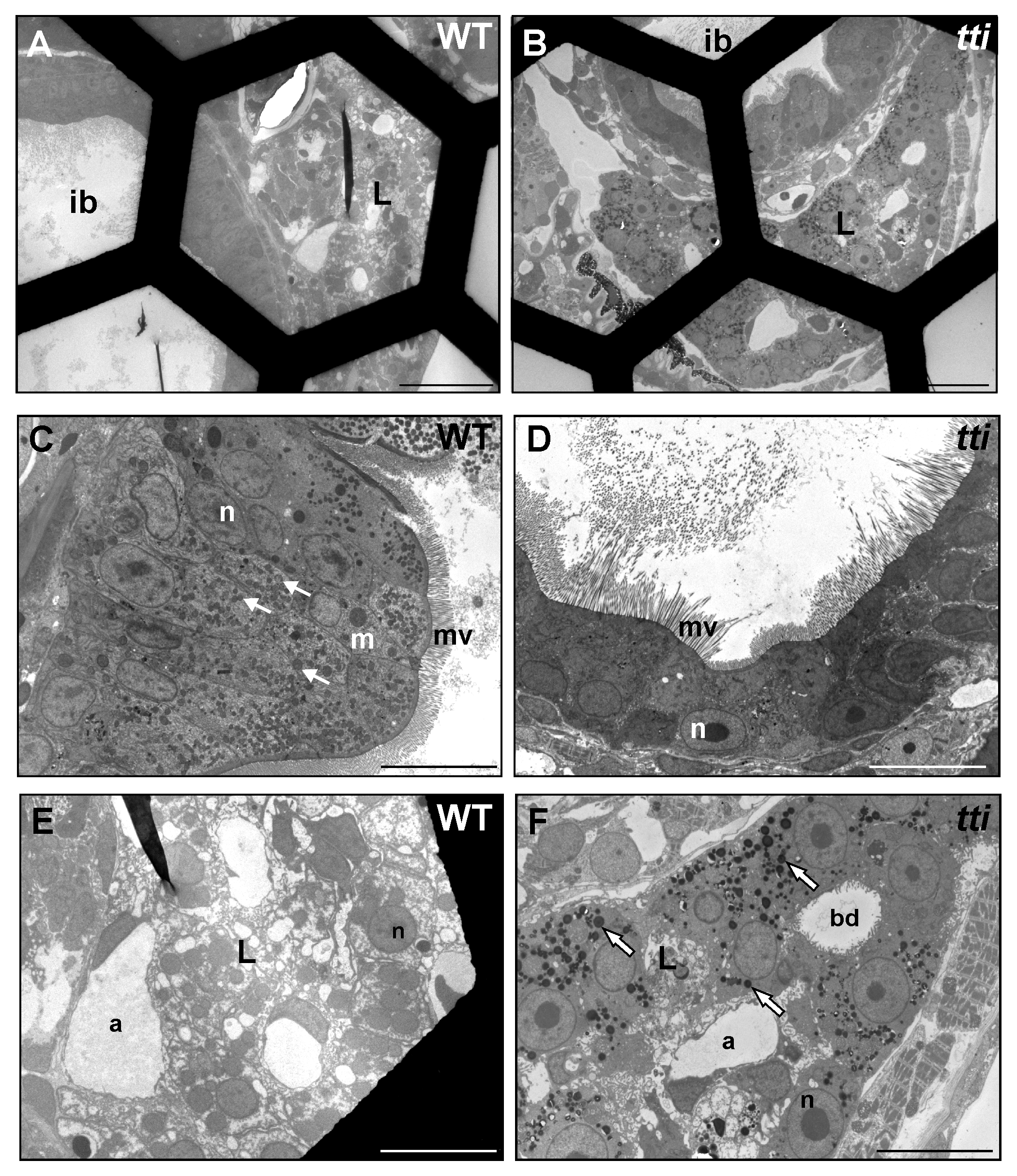

Supplement: Figure S6 — Absence of dead cells in the intestinal lumen of WT and ttis450 larvae at 7 dpf. (A–F) Transmission electron micrographs of transverse sections of WT and ttis450 larvae at 168 hpf (7 dpf). The number of conspicuous autophagosome-like structures in the IECs of ttis450 larvae has diminished by 7 dpf and there are no dead cells in the lumen (D). Meanwhile, liver cells of ttis450 larvae contain abundant autolysosome-like structures at this time-point (F, white arrows). Scale bars = 50 µm (A, B); 10 µm (C–F). ib, intestinal bulb; n, nucleus; m, mitochondria; mv, microvilli; l, liver; bd, bile duct; a, arteriole. (TIF) [file pgen.1003279.s006.tif]

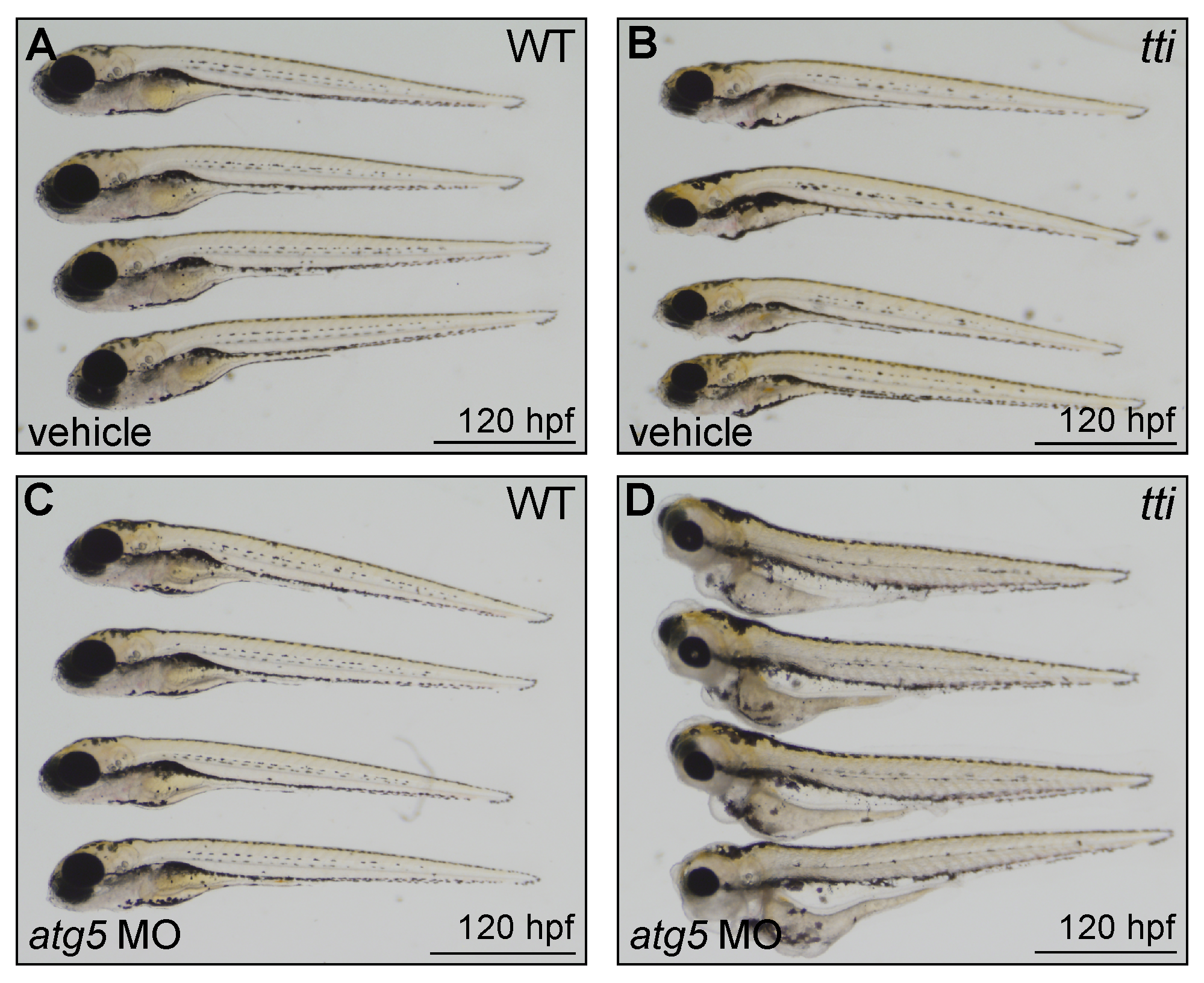

Supplement: Figure S7 — Disruption of autophagy in ttis450 larvae results in severe oedema. Upon microinjection into the yolk of 1–4 cell WT and ttis450 zebrafish embryos, an atg5-targeted MO (1 ng) produces severe oedema around the organs of ttis450 larvae at 120 hpf (D), while WT larvae are unaffected (C). WT and ttis450 larvae injected at the 1–4 cell stage with vehicle (A, B) are also unaffected. (TIF) [file pgen.1003279.s007.tif]

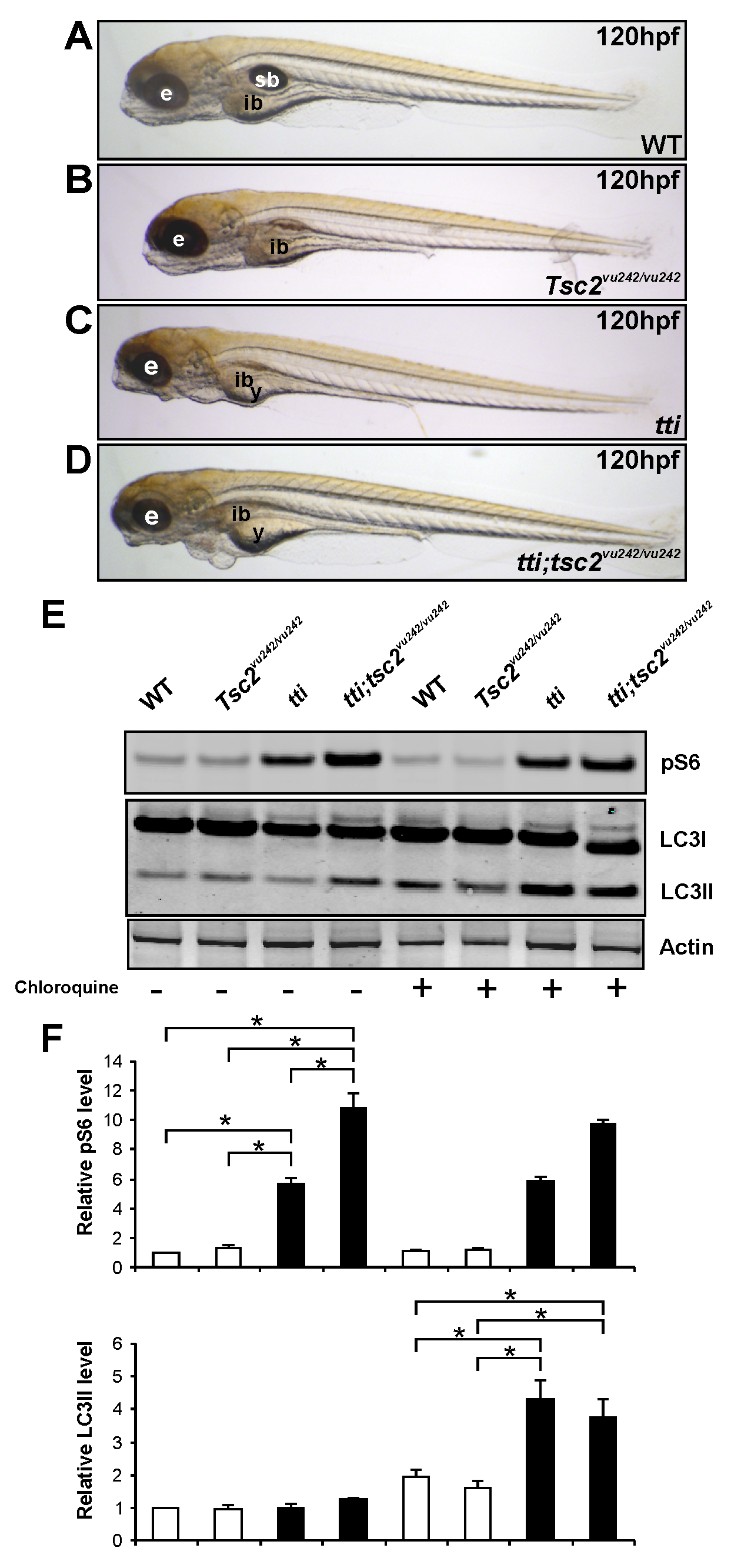

Supplement: Figure S8 — Autophagic flux in ttis450 larvae is not abrogated by Tor pathway activation. (A–D) Enhancing Torc1 activity by ablating Tsc2 activity in ttis450 larvae does not change their gross morphology at 120 hpf. Compound mutants (ttis450;Tsc2vu242/vu242) (D) are essentially indistinguishable from ttis450 larvae (C). Other panels show WT (A) and Tsc2vu242/vu242 mutant (B) larvae. (E,F) Western blot analysis of p-RPS6 and LC3 demonstrates that ttis450;Tsc2vu242/vu242 compound mutants at 96 hpf contain higher levels of p-RPS6 than ttis450 mutants due to increased Tor activity, yet LC3II levels are comparable between the two genotypes (refer to right hand half of the Western blot, where the larvae were pre-treated with chloroquine). p-RPS6 and LC3II levels are not significantly different between WT and tsc2vu242/vu242 larvae in the presence of chloroquine. Actin was used as a loading control. The levels of LC3II were quantitated by densitometric analysis of three independent Western blots. Data are represented as mean +/− SD, *p<0.05. (TIF) [file pgen.1003279.s008.tif]
