# Supplementary material for: Autophagy Induction Is a Tor- and Tp53-Independent Cell Survival Response in a Zebrafish Model of Disrupted Ribosome Biogenesis
Source: PLoS Genet. 2013 Feb 7;9(2):e1003279. doi: 10.1371/journal.pgen.1003279 (PMC3567153; doi:10.1371/journal.pgen.1003279)
Supplement: Text S1 — Sequences of primers and morpholinos and additional antibody information. (PDF) [file pgen.1003279.s009.pdf]

Text S1 - Sequences of primers and morpholinos and additional antibody information.

Primer sequences

To generate *pwp2h* riboprobes for wholemount *in situ* hybridization the following pair of primers was used: Forward: 5′*aattaaccctcactaaaggg*TACTCAGCGGATGGTGAATCG (T3 sequence in italics); Reverse: 5′*taatacgactcactataggg*AGAGGTGATGTACTGGCTT (T7 sequence in italics).

To genotype *tti*^{s450} and WT larvae the following pair of primers was used to generate amplicons containing the *Eco*N1 RFLP:

Forward (F): 5′AGAGGATTTCGGCCATTTCT;

Reverse (R): 5′ACCACTGTTGGTGTTCCACA

Primers used for qRT-PCR were as follows:

p21**:** F: 5′AGCTGCATTCGTCTCGTAG; R: 5′CGGTTGAAATAAAAACGGAATA

cyclinG1**:** F: 5′GTGCGGAGACGTTTTCCTT; R: 5′AAGACAGATGCTTGGGCTGA

mdm2**:** F: 5′TGACAACGAGAAACTGGTAAGA; R: 5′AAACATAACCTCCTTCATGGT

ΔN113p53: F: 5′ATATCCTGGCGAACATTTGG; R: 5′ACGTCCACCACCATTTGAAC

Primers used to generate a cDNA encoding mCherry fused to the N-terminus of zebrafish LC3 were: F: 5’CCTTCGGAAAAGACATT; R: 5’GCCAAATGAATGAAAGTTCA

Morpholino sequences

The sequence of the *pwp2h* morpholino was: 5’CTTGTACGCAAACTTCATATTTTCT.

The sequence of the *atg5* morpholino was: 5’CATCCTTGTCATCTGCCATTATCAT.

Antibodies

For Western blot analyses the primary antibodies used were: anti-phospho-RPS6 ($Ser^{240/244}$, #2215, Cell Signaling Technology, 1:1000), anti-RPS6 (#2217, Cell Signaling Technology, 1:1000) anti-LC3B (ab51520, Abcam, 1:1000) and anti-Actin (#A2066, Sigma-Aldrich; 1:2000). The anti-zebrafish Tp53 antibody was a kind gift of Sir David Lane (A*STAR, Singapore) and was diluted to 1:1000. Polyclonal secondary antibodies were IRDye800-conjugated goat anti-rabbit (#926-32211, LI-COR Biosciences; 1:15,000) for p-RPS6, LC3 and Actin and goat anti-mouse immunoglobulins/HRP (#2015-12, Dako; 1:40,000) for Tp53. For immunocytochemical analysis of wholemount intestinal sections, Alexa Fluor® 488 Dye (Life Technologies; 1:500), Rhodamine-Phalloidin (Jomar Biosciences; 1:250) and Hoechst (Sigma Aldrich; 1:500) were used to stain LC3, F-actin and DNA, respectively.
